# Biophysical characterization of the phase separation of TDP-43 devoid of the C-terminal domain

**DOI:** 10.1186/s11658-024-00615-4

**Published:** 2024-07-13

**Authors:** Tommaso Staderini, Alessandra Bigi, Clément Lagrève, Isabella Marzi, Francesco Bemporad, Fabrizio Chiti

**Affiliations:** 1 Department of Experimental and Clinical Biomedical Sciences “Mario Serio”, University of Florence, 50134 Florence, Italy; 2 Present Address: Department of Chemistry “Ugo Schiff”, University of Florence, 50019 Florence, Italy; 3 Present Address: Magnetic Resonance Center (CERM), University of Florence, Sesto Fiorentino, 50019 Florence, Italy; 4 Present Address: Chimie ParisTech-PSL, École Nationale Supérieur de Chimie de Paris, 11 rue Pierre et Marie Curie, 75231 Paris, France

**Keywords:** Liquid–liquid phase separation, LLPS, Liquid–solid phase separation, Self-assembly, Motor neuron diseases, Electrostatics, RNA-binding proteins

## Abstract

**Background:**

Frontotemporal lobar degeneration with ubiquitin-positive inclusions (FTLD-TDP), amyotrophic lateral sclerosis (ALS) and limbic-predominant age-related TDP-43 encephalopathy (LATE) are associated with deposition of cytoplasmic inclusions of TAR DNA-binding protein 43 (TDP-43) in neurons. One complexity of this process lies in the ability of TDP-43 to form liquid-phase membraneless organelles in cells. Previous work has shown that the recombinant, purified, prion-like domain (PrLD) forms liquid droplets in vitro, but the behaviour of the complementary fragment is uncertain.

**Methods:**

We have purified such a construct without the PrLD (PrLD-less TDP-43) and have induced its phase separation using a solution-jump method and an array of biophysical techniques to study the morphology, state of matter and structure of the TDP-43 assemblies.

**Results:**

The fluorescent TMR-labelled protein construct, imaged using confocal fluorescence, formed rapidly (< 1 min) round, homogeneous and 0.5–1.0 µm wide assemblies which then coalesced into larger, yet round, species. When labelled with AlexaFluor488, they initially exhibited fluorescence recovery after photobleaching (FRAP), showing a liquid behaviour distinct from full-length TDP-43 and similar to PrLD. The protein molecules did not undergo major structural changes, as determined with circular dichroism and intrinsic fluorescence spectroscopies. This process had a pH and salt dependence distinct from those of full-length TDP-43 and its PrLD, which can be rationalized on the grounds of electrostatic forces.

**Conclusions:**

Similarly to PrLD, PrLD-less TDP-43 forms liquid droplets in vitro through liquid–liquid phase separation (LLPS), unlike the full-length protein that rather undergoes liquid–solid phase separation (LSPS). These results offer a rationale of the complex electrostatic forces governing phase separation of full-length TDP-43 and its fragments. On the one hand, PrLD-less TDP-43 has a low pI and oppositively charged domains, and LLPS is inhibited by salts, which attenuate inter-domain electrostatic attractions. On the other hand, PrLD is positively charged due to a high isoionic point (pI) and LLPS is therefore promoted by salts and pH increases as they both reduce electrostatic repulsions. By contrast, full-length TDP-43 undergoes LSPS most favourably at its pI, with positive and negative salt dependences at lower and higher pH, respectively, depending on whether repulsive or attractive forces dominate, respectively.

**Supplementary Information:**

The online version contains supplementary material available at 10.1186/s11658-024-00615-4.

## Introduction

Amyotrophic lateral sclerosis (ALS), half of the cases of frontotemporal lobar degeneration (FTLD) and the recently discovered limbic-predominant age-related TDP-43 encephalopathy (LATE) are associated with a common histopathology based on the presence of cytoplasmic inclusions of the nuclear TAR DNA-binding protein 43 (TDP-43) in neurons and motoneurons of the central nervous system, accompanied by a concomitant disappearance of the soluble protein from the nucleus of the same cells [1–5]. The cytoplasmic inclusions are either diffuse/granular, skein-like or round in morphology and the constituent TDP-43 protein molecules are polyubiquitinated, hyperphosphorylated, in part fragmented with a significant presence of C-terminal fragments, acetylated, SUMOylated, citrullinated and monomethylated [1–3, 6–10], with fragmentation observed particularly in the brain and more rarely in the spinal cord in ALS patients [11].

ALS and FTLD-TDP are characterized by a combination of loss-of-function (LOF), originating from the impoverishment of functional TDP-43 in the nucleus, and a gain-of-function (GOF), resulting from the formation of cytoplasmic TDP-43 assemblies that act as saboteurs with consequent dysfunction of cellular activities [12–16]. TDP-43 inclusions are also frequently found in the brain of Parkinson disease [17], Alzheimer disease [18], Huntington disease [19], Creutzfeldt-Jacob disease [20], chronic traumatic encephalopathy [21] and others, indicating that this protein has a high propensity to aggregate in a large spectrum of neurodegenerative conditions in which the proteostasis network (PN) is compromised.

TDP-43 contains 414 amino acid residues and consists of an N-terminal domain (NTD_1–76_), two RNA recognition motifs (RRM1_106–176_ and RRM2_191–259_) and a C-terminal domain (CTD_274–414_), also called low-complexity domain (LCD) or prion-like domain (PrLD). This latter domain is intrinsically disordered with an amphipathic helix spanning approximately amino acid residues 320–340 [22, 23], whereas the remaining three domains are folded and their structures have been solved with X-ray crystallography or nuclear magnetic resonance (NMR) spectroscopy [14, 24, 25]. The TDP-43 sequence also includes a nuclear localization signal (NLS_82–98_) and a nuclear export signal (NES_239–250_), which allow the protein to shuttle between the nucleus and the cytosol, although in non-pathological conditions the nuclear population is by far the most represented [26]. TDP-43 was first proposed to be dimeric through interactions involving NTDs of two different molecules [27–31]. Later on, however, isolated NTDs, and consequently full-length TDP-43 molecules, were found to form head-to-tail interactions leading to a propagation of the functional oligomeric state well ahead of a dimer [24, 25].

The conversion of native, soluble, dimeric/oligomeric TDP-43 into pathological, solid-phase inclusions is still an unclear process. One of the complexities of this process is the ability of TDP-43 to form liquid droplets in cells in which TDP-43, as well as other proteins and RNA molecules, adopt a liquid phase separated from the bulk cytosolic solution, through a process known as liquid–liquid phase separation (LLPS) [32–39]. Several nuclear and cytosolic membraneless organelles have been shown to contain TDP-43 [39], including stress granules [32, 33], paraspeckles [34, 35], stress nuclear bodies [36], cytosolic droplets independent of stress granules [37] and anisosomes [38]. Solid inclusions of TDP-43 have been observed to form within cytosolic stress granules in a liquid-to-solid transition [37, 40], but have also been observed to form independently of these liquid organelles [40–43].

To explore the mechanisms through which TDP-43 undergoes LLPS, several studies have been carried out in vitro, using purified recombinant TDP-43 or its fragments. To this aim, considerable effort has been expended using the purified PrLD, which has been found, by many different investigators working independently of each other, to form in vitro bona fide liquid droplets that have a round morphology, ability to coalesce and the dynamic behaviour typical of liquids, as assessed with fluorescence recovery after photobleaching (FRAP) [22, 23, 44–47]. By contrast, LLPS has not been generally observed for the pure full-length protein, which has been found, again by many different independent investigators and under different solution conditions, to self-assemble rapidly into species unable to coalesce into larger round droplets and exhibiting weak or no recovery of relative fluorescence intensity (*RFI*) after photoblaching [25, 48–52]. LLPS was observed only occasionally for pure full-length TDP-43 and only when it was fused to large solubilizing protein tags, such as the small ubiquitin-like modifier (SUMO) or the maltose-binding protein (MBP) [53]. However, also in the presence of large tags, LLPS was not generally observed for the full-length protein [25, 48, 49, 51]. Surprisingly, genuine LLPS was observed for a purified TDP-43 construct containing the first three folded domains (residues 1–273) and devoid of the PrLD, which was named PrLD-less TDP-43 [54]. Nevertheless, LLPS of this three-domain construct has been studied only in one paper and was shown to have a dependence on various solution parameters distinct from that observed for the PrLD LLPS [54], therefore requiring experimental confirmation and a detailed multi-parametric analysis of the solution conditions governing the LLPS process of this construct. Moreover, a LLPS behaviour of PrLD-less TDP-43 in vitro is, in principle, surprising following the presence and absence of the same behaviour in the PrLD of TDP-43 and full-length protein, respectively.

Here, we have purified recombinant PrLD-less TDP-43 containing amino acid residues 1–260 and devoid of large tags and have studied its self-assembly using an array of biophysical techniques to study the morphology of the assemblies, their state of matter and the structure of PrLD-less TDP-43 molecules adopted within them under different solution conditions. We will show that this protein construct forms round assemblies able to coalesce with time into larger and still round assemblies and exhibiting significant recovery of *RFI* after photobleaching in at least a fraction of them, showing a behaviour distinct from full-length TDP-43 and more similar to the purified PrLD. We will also show that the pH and salt dependence of LLPS of PrLD-less TDP-43 is distinct from that of the TDP-43 PrLD and also from that found in liquid–solid phase separation (LSPS) of the full-length protein, providing a rationale of the forces governing phase separation of full-length TDP-43 and its two fragments.

## Materials and methods

### PrLD-less TDP-43 cloning, expression and purification

We obtained the construct encoding PrLD-less TDP-43 starting from a pNic28-Bsa4 plasmid containing the sequence of full-length TDP-43 with a tag of six histidine residues at the N-terminus, using PCR and the QuikChange XL Site-Directed Mutagenesis Kit (Agilent Technologies). Forward primer 5^I^-CGTTCATATATCCAATGCCTAACCTAAGCACAATAGCAAT- ^I^3 and reverse primer 5^I^-ATTGCTATTGTGCTTAGGTTAGGCATTGGATATATGAACG-^I^3, were used to introduce the stop codon at nucleotides 779–781 (corresponding to amino acid residue 261). The PCR product was quantified with NanoDrop One (Thermo Fisher Scientific). The resulting pNIC28-Bsa4 plasmid, coding for the PrLD-less TDP-43 sequence (residues 1–260), was transformed into *Escherichi coli* NEB DH5α competent cells. The colonies were plated, cultured and the plasmid was extracted and sequenced (BMR Genomics, Padua, Italy) to confirm the presence of the desired stop codon.

Protein expression and purification was carried out as described previously [55] with a few modifications. In brief, *E. coli* C41(DE3) cells (New England Biolabs) were transformed with the plasmid, grown overnight at 37 °C in 20 g/L Luria–Bertani (LB) medium containing 50 μg/mL kanamycin. Then, 25 mL of the resulting medium were diluted into 1 L of fresh LB medium, supplemented with 50 μg/mL kanamycin and grown at 37 °C until OD_600_ was 0.7. The culture medium was cooled down to 4 °C for 30 min and expression of the protein was induced with 0.5 mM isopropyl β-d-1-thiogalatoside (IPTG) at 18 °C overnight. Bacteria were then harvested at 6790*g* for 30 min at 4 °C, resuspended in lysis buffer [50 mM sodium phosphate, pH 8.0, 300 mM NaCl, 5 mM imidazole, 1 mM phenylmethanesulfonyl fluoride (PMSF), 5 mM dithiothreitol (DTT)] and then sonicated at 30 W and 20 kHz (6 cycles, 20 s on/off). The soluble protein was recovered by centrifugation at 27,000*g* for 30 min at 4 °C and DNA was then precipitated adding 10 mg/mL of streptomycin sulfate, under stirring at 4 °C, followed by a new centrifugation at 27,000*g* for 30 min at 4 °C. The supernatant was loaded onto 10 mL of HisPur Ni–nitrilotriacetic acid (Ni–NTA) resin (Thermo Fisher Scientific) in a gravity flow column pre-equilibrated with 50 mM sodium phosphate, pH 8.0, 300 mM NaCl, 5 mM imidazole, 25 °C. The column was washed with the same buffer and two elution steps were carried out with 50 mM sodium phosphate pH 8.0, 300 mM NaCl, 75 mM imidazole, 5 mM DTT and the same buffer containing 250 mM imidazole, respectively. The eluted protein sample was dialysed overnight at 4 °C to remove imidazole and the day after it was diluted five times with 50 mM sodium phosphate, pH 8.0, 5 mM DTT, for weak anionic ion exchange chromatography using a 5 mL HiTrap DEAE FF column pre-equilibrated at 4 °C with the same buffer, with an Akta Pure 25L system (GE Healthcare). The protein was eluted with a gradient step method using 50 mM sodium phosphate, pH 8.0, 1 M NaCl, 5 mM DTT. The sample containing PrLD-less TDP-43 was concentrated at 4 °C using Amicon Ultra centrifugal filter units (Millipore Sigma) with 10 kDa molecular weight cut-off (MWCO), down to around 10 mL and subjected to size exclusion chromatography (SEC) using a Hi Load 26/60 Superdex 75 pg column (GE Healthcare) pre-equilibrated at 4 °C with 20 mM Tris–HCl, pH 8.0, 300 mM NaCl, 5 mM DTT. The elution was carried out isocratically at a flow rate of 1.5 mL/min and the fractions containing PrLD-less TDP-43 protein were merged, concentrated at 4 °C using Amicon Ultra centrifugal filter units with 10 kDa MWCO down to ca. 1–2 mL. The concentrated sample (1.5–1.6 mg/mL) was finally stored at −20 °C in the same buffer used for size exclusion chromatography (SEC), as described above.

Protein concentration was determined by optical absorption spectroscopy using a molar extinction coefficient at 280 nm (ε_280_) of 28,420 M^−1^ cm^−1^. The final purified protein had the 22-residue stretch MHHHHHHSSGVDLGTENLYFQS at the N-terminus before Met1, contained 282 residues and had a molecular weight (MW) of 32,053 Da.

### Sodium dodecyl sulphate polyacrylamide gel electrophoresis (SDS–PAGE)

Protein samples were analysed by SDS–PAGE, using 4–20% (w/v) Mini-Protean TGX Precast Polyacrylamide gels (Bio-Rad). Prior to electrophoresis, the protein samples were concentrated by precipitation with cold acetone and the pellet dissolved in 30 µL of 50 mM Tris–HCl, pH 8.0, 300 mM NaCl, and 10 µL of 4× sample buffer and boiled for 5 min at 98 °C; 15 µL of each sample was subjected to the electrophoresis for ∼1 h at 150 V. Gels were stained with Coomassie brillant blue solution and destained with a solution of ethanol and acetic acid.

### Dynamic light scattering (DLS)

DLS analysis was performed with a Malvern Sizer Nano S DLS device (Malvern Panalytical), at 25 °C using a temperature controlled internal Peltier system. Size distribution was determined using a disposable low volume cuvette (50 μL) in backscatter (173°) method. Conditions were 43 μM PrLD-less TDP-43 in 20 mM Tris–HCl, pH 8.0, 300 mM NaCl, 5 mM DTT, 25 °C. Viscosity and refractive index were set on the instruments at 0.9494 cp and 1.335, respectively.

### Size exclusion chromatography (SEC)

SEC was carried out using a Superdex 200 Increase 10/300 GL column (GE Healthcare) equilibrated with 20 mM Tris–HCl, pH 8.0, 300 mM NaCl, 5 mM DTT, at 4 °C and an Akta Pure 25L system. PrLD-less TDP-43 and standard samples with known MW, such as apoferritin (443 kDa), bovine serum albumin in monomeric form (66 kDa) and dimeric form (132 kDa), carbonic anhydrase (29 kDa) and lysozyme (14.4 kDa), were solubilized in the same buffer of PrLD-less TDP-43 and then loaded in volumes of 100 µL each. Elution volumes (*V*_*e*_) were taken as the volumes of buffer passed through the column between sample injection and attainment of highest absorbance. A plot of log[*MW*] versus *V*_*e*_ was obtained with the five standard data points and fitted to a linear regression curve. The *V*_*e*_ measured for PrLD-less TDP-43 (injected at 15.6 µM) was then interpolated into the resulting standard curve (*y* axis) to obtain the corresponding *MW* value (*x* axis), according to the previously described method [56], which is widely used to discriminate dimers from monomers of proteins of interest [57–59].

### Differential scanning fluorimetry (DSF)

DSF was performed using a 7500 Real-Time PCR machine (Bio-Rad CFX) by selecting the fluorescein amidite filter. Then, 49 µL of 40 µM PrLD-less TDP-43 was mixed with 1 µL of Sypro Orange dye 250x (Thermo Fisher Scientific). 45 µL of protein–dye solution were dropped off (15 µL per well) in a MicroAmp Fast Optical 96-well reaction plate, sealed with a MicroAmp adhesive film (Thermo Fisher Scientific). The samples were heated with a ramp speed, consisting of a 2 min pause at 25 °C, followed by a gradient temperature of 1 °C until 95 °C. The curve was obtained by plotting Sypro Orange fluorescence versus temperature and averaging over three curves. The resulting curve was converted into its first derivative to get the apparent melting temperature as a positive peak.

### Induction of PrLD-less TDP-43 self-assembly

A PrLD-less TDP-43 sample was thawed and spun down at 18,000*g*, 4 °C, for 15 min to remove possible aggregates. The supernatant was concentrated with Amicon Ultra 0.5 mL centrifugal filter units with 10 kDa MWCO and desalted with a Sephadex G15 resin column (Pharmacia Fine Chemicals) in 20 mM Tris–HCl, pH 8.0. Protein concentration was determined with NanoDrop One and adjusted to 10 μM by dilution in 20 mM Tris–HCl, pH 8.0. Protein self-assembly was induced by diluting the protein 1:1 into 10 mM acetate buffer, pH 5.0, 10% (w/v) poly(ethylene glycol) 8000 (PEG 8000), 2 mM Tris(2-carboxyethyl)phosphine (TCEP), 25 °C, containing either 0 mM or 300 mM NaCl (final conditions were 5 µM PrLD-less TDP-43, pH 5.5, 0 mM or 150 mM NaCl, 5% (w/v) PEG 8000, 1 mM TCEP, 25 °C) and incubating the resulting PrLD-less TDP-43 sample under shaking at 560 rpm on a TS-100 Thermo Shaker at 25 °C for 2 h. To investigate the effects of the solution conditions on PrLD-less TDP-43 self-assembly, a multi-parametric analysis was carried out, where 24 phase separation buffers were designed as subtle variations of our standard conditions reported above, in which pH, NaCl and percentage (w/v) of PEG 8000 were varied one by one, as indicated.

### Confocal fluorescence microscopy

A PrLD-less TDP-43 sample was thawed and spun down at 18,000*g*, 4 °C, for 15 min to remove possible aggregates. It was then labelled (40 μM) at 4 °C overnight or at room temperature for 2 h in the dark on a mechanical shaker with tetramethylrhodamine-5-maleimide (TMR) (Invitrogen) in a dye:protein ratio of 1:10, using the same buffer in which the protein was initially stored (20 mM Tris–HCl, pH 8.0, 300 mM NaCl, 5 mM DTT). The sample was then concentrated, desalted, diluted and treated to induce self-assembly as described in the previous section [final conditions were 5 µM PrLD-less TDP-43, pH 5.5, 0 mM or 150 mM NaCl, 5% (w/v) PEG 8000, 1 mM TCEP, 25 °C]. An aliquot was also concentrated, desalted and diluted to maintain it in a native state without phase separation (final conditions were 5 µM PrLD-less TDP-43, 20 mM Tris–HCl, pH 8.0, 25 °C). Then, 5 μL of protein samples were spotted at different timepoints (0, 5, 10, 60 min) onto a clean microscope slide, covered with a 12 mm glass coverslip and visualized using a TCS SP8 scanning confocal fluorescence microscopy system (Leica Microsystems) equipped with an argon laser source at 514 nm. TMR was excited at 514 nm and fluorescence emission was collected at 550–700 nm.

### Fluorescence recovery after photo-bleaching (FRAP)

A PrLD-less TDP-43 sample was thawed and spun down at 18,000*g*, 4 °C, for 15 min to remove possible aggregates. It was then labelled (40 μM) in the dark at 4 °C overnight on a mechanical shaker with Alexa Fluor 488 (AAT Bioquest) in a 1:10 ratio (dye:protein) in the same buffer in which the protein was initially stored (20 mM Tris–HCl, pH 8.0, 300 mM NaCl, 5 mM DTT). After labelling, non-reacted dye was removed by passing the reaction mixture through a Sephadex G15 resin column. The sample was treated to induce self-assembly as described in the previous section and 40 μL of the protein sample were spotted into a well of a μ-slide 18-well glass-bottom sterilized plate (Ibidi GmbH). The FRAP experiment was performed using the LAS AF FRAP Application Wizard on a Leica TCS SP8 3X microscope equipped with a Leica HC PL APO CS2 100x/1.40 oil white objective and the 488-nm laser, as reported previously [52]. Briefly, the experiment involved two pre-bleaching frames, and photobleaching was then performed in regions of interest (ROIs) at 100% laser intensity for 15.6 s (20 iterations, one frame every 0.78 s). Fluorescence recovery was recorded over a period of 145 s (one frame every 5 s) at 2.1% laser intensity to avoid unintentional photobleaching over the post-bleach period. To optimize the scan speed, images were acquired at a speed of 700 Hz in bidirectional mode. The FRAP experiment was conducted for a maximum of 20 min from solution jump, then the sample was prepared again.

The relative fluorescence intensity (*RFI*) was reported from a value of 0.0 (minimal post-bleaching intensity) to a value of 1.0 (pre-bleaching intensity) and plotted versus the time elapsed after the end of bleaching (taken as time 0 s), and the resulting kinetic plots were analysed with a procedure of best fitting using a double exponential function of the form:

1 $${\text{RFI}}\left( t \right) = {\text{RFI}}({\text{eq}}) {-}A_{{{\text{fast}}}} {\text{exp}}\left( { - k_{{{\text{fast}}}} {\text{t}}} \right){-}A_{{{\text{slow}}}} {\text{exp }}\left( { - k_{{{\text{slow}}}} {\text{t}}} \right)$$

where RFI(*t*) is the relative fluorescence intensity at time *t*, RFI(*eq*) is the relative fluorescence intensity at the apparent equilibrium (time ∞), *A*_fast_ and *A*_slow_ are the amplitudes of the fast and slow exponential fluorescence changes, respectively, and *k*_fast_ and *k*_slow_ are the fast and slow rate constants, respectively, expressed in s^−1^.

### Turbidimetry

Turbidimetry was assessed using an ultrafast Synergy H1 plate reader (BioTek), in non-binding, chimney style, flat clear bottom 96-well microplates with lid (Greiner, Bio-One). PrLD-less TDP-43 self-assemblies were induced under various experimental conditions, as described above, shaking in continuous way at 567 counts per min for 2 h at 25 °C. The turbidity was detected every 13 s at 600 nm (OD_600_). The measurements were performed in triplicates (*n* = 3) for each sample and condition, blank subtracted and then averaged. Plots of turbidity versus time were obtained by considering a window of 120 s for the first five points, a window of 600 s for the following four points and 1800 s for the last two points, averaging all values recorded within that window and reporting a mean value centered in the same window.

### Far-UV circular dichroism (CD) spectroscopy

Far-UV CD spectra were recorded for native and unfolded purified PrLD-less TDP-43 to determine their secondary structures. Conditions were 3.5 µM PrLD-less TDP-43, in 20 mM Tris–HCl, pH 8.0, 300 mM NaCl, 5 mM DTT, 25 °C for the native state and 3.5 µM PrD-Less TDP-43, in 20 mM Tris–HCl, pH 8, 8 M urea, 25 °C for the unfolded state. The unfolded spectrum was recorded until 212 nm due to the high tension (HT) voltage at lower wavelengths. Far-UV CD spectra were also recorded from 260 to 200 nm on PrLD-less TDP-43 during self-assembly. Final sample conditions were as reported above in the dedicated subsection. Spectra were recorded using a Jasco J-810 Spectropolarimeter equipped with a thermostated cell holder attached to a Julabo 200F water bath and using 1 mm pathlength quartz cuvettes (Hellma). Spectra were averaged from eight scans, blank-subtracted and normalized to mean residue ellipticity.

### Intrinsic fluorescence spectroscopy

Intrinsic fluorescence spectra were recorded for native and unfolded purified PrLD-less TDP-43 to determine their structures in terms of tryptophan chemical environment. Conditions were 3.5 µM PrLD-less TDP-43, in 20 mM Tris–HCl, pH 8.0, 300 mM NaCl, 5 mM DTT, 25 °C for the native state and 3.5 µM PrD-Less TDP-43, in 20 mM Tris–HCl, pH 8, 8 M urea, 25 °C for the unfolded state. Intrinsic fluorescence spectra were also recorded on PrLD-less TDP-43 during self-assembly. Final sample conditions were the same as reported above in the dedicated subsection. Spectra were recorded using a Cary Eclipse spectrofluorometer (Agilent Technologies) equipped with a thermostated cell holder attached to an Agilent PCB 1500 water Peltier system, from 290 to 500 nm at 280 nm excitation, using a 10 × 2 mm quartz cuvette (Hellma) for the native and unfolded PrLD-less TDP-43 samples and a 3 × 3 small volume quartz cuvette (Hellma) for the self-assembled PrLD-less TDP-43 experiments. Excitation and emission slits were 5 nm in both cases.

## Results

### PrLD-less TDP-43 is pure and folded

The gene coding for PrLD-less TDP-43 (residues 1–260 plus a His-tag at the N-terminus) was subcloned into a pNIC28-Bsa4 plasmid, recombinantly expressed in *E. coli* C41(DE3) cells and purified as described in the Materials and methods section. The final conditions of the purified protein construct were 20 mM Tris–HCl, pH 8.0, 300 mM NaCl, 5 mM DTT. The domain composition of PrLD-less TDP-43, along with positions of cysteine residues used for labelling and tyrosine/tryptophan residues detected with fluorimetry, are reported in Fig. S1. The six cysteine residues, two per domain, are reduced in the native protein and kept reduced in our experiments with DTT or TCEP (Fig. S1). The protein harbours seven tyrosine residues, with four, two and one located in the NTD, RRM1 and RRM2, respectively. One tryptophan is in the NTD and two other in the RRM1 (Fig. S1). All of them are at least partially buried in the hydrophobic core, causing a red-shift of their intrinsic fluorescence upon unfolding.

Prior to the analysis, the protein was checked for high purity, proper fold and size. SDS–PAGE revealed a single, highly intense band with a MW of ~32 kDa, in agreement with the expected theoretical MW of 32.053 kDa (Fig. S2A). Analytical SEC showed a main peak with an elution volume of 14.8 mL (Fig. S2B). By plotting this value into a calibration curve obtained with apoferritin, bovin serum albumin in monomeric and dimeric forms, carbonic anhydrase and lysozyme, which are proteins of known MW, the MW of PrLD-less TDP-43 was found to be ~63.8 kDa, close to the theoretical MW of a construct of this size with dimeric fold, that is 64.106 kDa (Fig. S2B). However, given the complex relationship between elution volume, protein molecular weight and protein geometry, we cannot exclude that the peak arises from a monomer or higher order oligomer.

DLS analysis showed the presence of a single peak with an apparent hydrodynamic diameter (*D*_*h*_) of 79 ± 4 Å (Fig. S2C). The expected *D*_*h*_ values for a TDP-43 construct of this size (282 residues) and containing three folded domains of known diameter, are 54.4 Å, 72.5 Å and > 100 Å for monomeric, dimeric and higher-order oligomeric forms, respectively, as determined using the previously described approach based on DLS theory [60]. In particular, any hypothetical spatial arrangement of the three folded domains in a monomer would lead to a *D*_*h*_ value lower than 79 ± 4 Å. The DLS analysis, therefore, indicates that PrLD-less TDP-43 is mainly a dimer. Large aggregates were not detectable with SEC and DLS, indicating that they were absent (Fig. S2B,C).

The far-UV CD spectrum showed a minimum at 208 nm and a shoulder at 222 nm, suggesting the presence of a significant α-helical structure (Fig. S2D). The spectrum did not exclude the presence of β-sheet content, not quite for the presence of a minimum in the 215–220 nm range, that is likely obscured by the α-helical contribution, but because of a positive peak closer to the peak expected for β-sheet (196 nm) than for α-helix (192 nm), as previously reported [61]. This spectrum is consistent with the presence of α-helices and β-sheets in all three NTD, RRM1 and RRM2 domains [14, 24, 25, 62]. The spectrum shown here is fully consistent with that shown previously for the same TDP-43 construct [61, 63] and significantly different from that shown previously for full-length TDP-43, which showed a less negative and more negative mean residue ellipticity at 208–230 nm and 190–205 nm, respectively [60], in agreement with the additional presence of the extended PrLD and, consequently, a higher fraction of disordered secondary structure.

The intrinsic fluorescence spectrum showed a single but broad peak in the range of 330–350 nm, with the maximum value at 340 nm (Fig. S2E), indicating that the protein is folded, as tryptophan residues of folded and unstructured proteins have maxima of fluorescence emission at 320–345 nm and 350–357 nm, respectively [64]. Moreover, the DSF technique, which allows the sensitive assessment of protein stability and folding, revealed a curve with an evident structural transition and an apparent melting temperature (*T*_*m*_) of 48.5 °C, confirming that purified native PrLD-less TDP-43 is folded and stable at temperature values lower than the *T*_*m*_ (Fig. S2F).

Hence, the data confirmed the purity, proper folding and dimeric structure of PrLD-less TDP-43.

### PrLD-less TDP-43 assemblies were round and coalesced with time

**Fig. 1:**
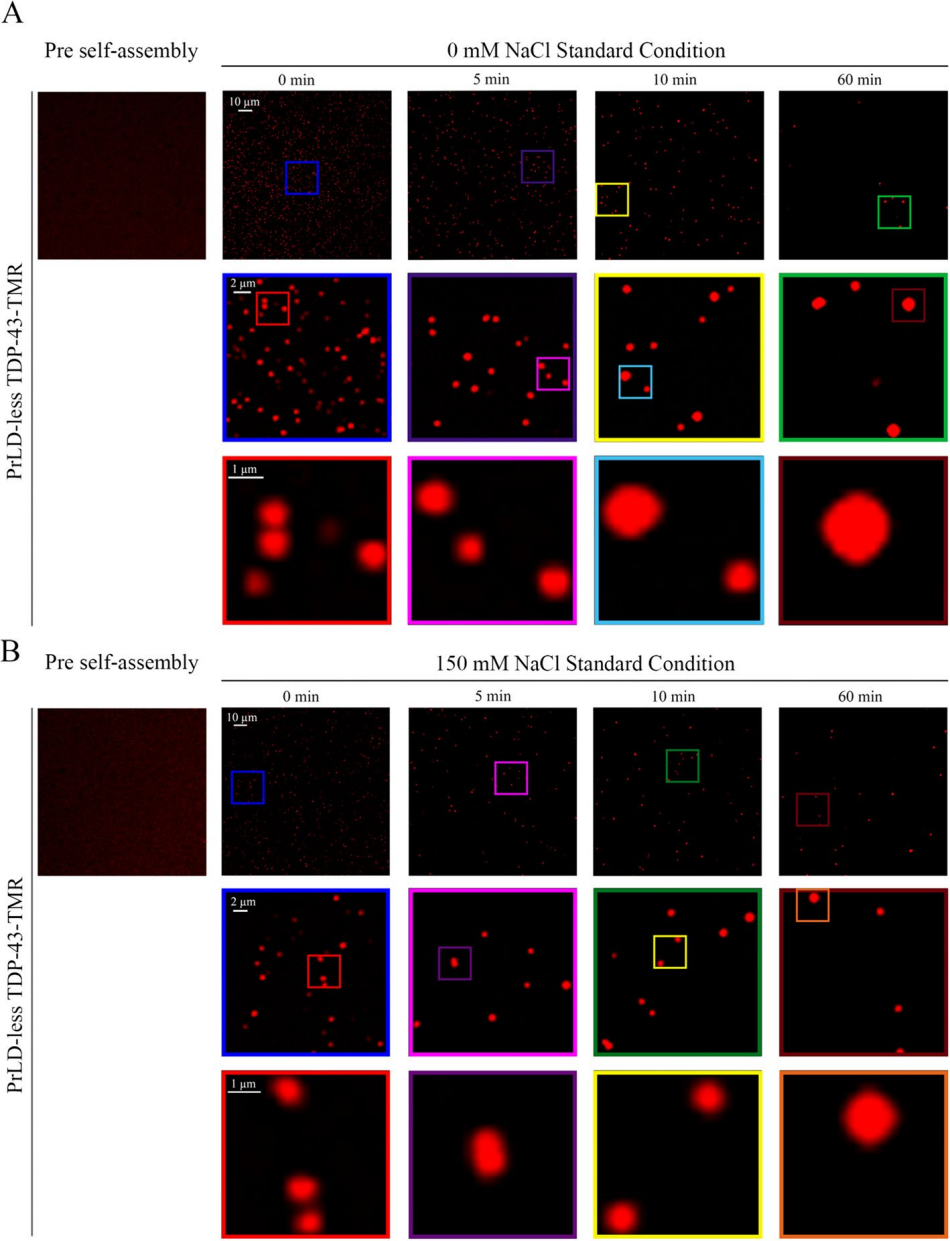
Confocal fluorescence microscopy images of PrLD-less TDP-43 assemblies. Representative confocal fluorescence images of PrLD-less TDP-43-TMR during self-assembly in 0 mM NaCl (**A**) and 150 mM NaCl (**B**). In the second and third rows, higher magnifications of the confocal fluorescence images are shown in the coloured boxed areas. The isolated images on the left were acquired with native PrLD-less TDP-43-TMR under conditions that do not promote phase separation

To assess the propensity of PrLD-less TDP-43 to phase separate in vitro and examine the size and morphology of its assemblies, the native protein sample was first labelled with TMR in 1:10 molar ratio (dye:protein), so that only one-tenth of the protein molecules were labelled in the sample. Confocal fluorescence images of PrLD-less TDP-43-TMR under native conditions (5 μM PrLD-less TDP-43-TMR, 20 mM Tris–HCl, pH 8.0, 25 °C) showed a diffused red fluorescence signal, indicating the absence of assemblies or aggregates (Fig. 1, left images). The protein was then incubated under final conditions that promoted PrLD-less TDP-43 self-assembly: 5 μM PrLD-less TDP-43-TMR, pH 5.5, 0 mM or 150 mM NaCl, 5% (w/v) PEG 8000, 1 mM TCEP, 25 °C (see Materials and methods for more details). The term ‘phase separation’ is used here to mean the conversion of the protein from its soluble state into self-assemblies, regardless of their solid or liquid state of matter, referring to the generic term including both liquid–liquid and liquid–solid phase separations, as theorized previously [65].

Early confocal fluorescence images acquired at 0 min in both 0 mM and 150 mM NaCl revealed that PrLD-less TDP-43 phase separated rapidly with formation of round-shaped assemblies with a size of ~0.5–1.0 μm (Fig. 1). The diffuse fluorescence signal previously observed under native conditions was no longer visible in the background in both conditions, indicating that TMR-labelled PrLD-less TDP-43 completely partitioned into the assemblies. This is particularly evident with a quantification of the background fluorescence emissions before and after the solution changes (Fig. S3). At later timepoints, protein condensates maintained approximately, in both conditions, their round-shaped morphology, but appeared progressively larger at 5 min, 10 min and 60 min, up to ~2 µm of diameter (Fig. 1). Moreover, their density decreased progressively with time, again at both salt concentrations (Fig. 1). These observations indicate that the small assemblies formed early coalesce together to form larger droplets. Most importantly, the late assemblies diverged from those imaged in this laboratory for full-length TDP-43 under identical conditions of solution, labelling and protein concentration, which appeared to cluster together into large irregular species without coalescing [52]. They also diverged from those imaged in other laboratories for full-length TDP-43 under similar conditions, with the individual spherical species assembling together in chain-like species or in a more disordered fashion [25, 48, 49, 51]. Interestingly, during the time course, the species formed in 0 mM NaCl appeared slightly larger than in 150 mM NaCl, suggesting that droplet formation and further fusion is facilitated at low ionic strength, unlike full-length TDP-43 [49, 52, 53] and the isolated PrLD [22, 23, 45–47].

### A few PrLD-less TDP-43 assemblies had large fluorescence recovery after photobleaching

**Fig. 2:**
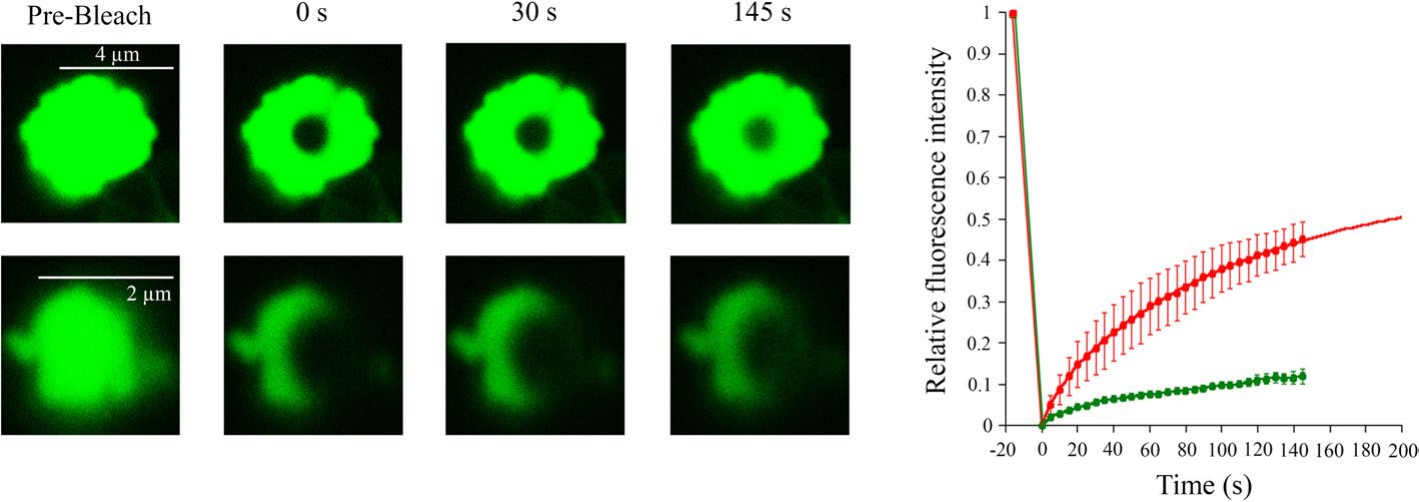
FRAP of PrLD-less TDP-43 assemblies. Representative confocal fluorescence images of 5 μM PrLD-less TDP-43 labelled with Alexa-488 during self-assembly in 150 mM NaCl, and recorded at time 0 min, as in Fig. 1. The figure shows, in particular, representative images from FRAP experiments on two distinct classes of assemblies (top and bottom panels, respectively). Relative fluorescence intensity (RFI) after photobleaching was plotted over time for 3 distinct assemblies (*n* = 3) of each class which were averaged (red and green curves, respectively). Fluorescence values were normalized to pre-bleach fluorescence values (taken as 1.0) and to post-bleach fluorescence values (taken as 0.0). Error bars: SEM. The red continuous line represents the best fit of experimental data to the double exponential equation (Eq. 1) described in the Materials and methods section

To investigate the molecular dynamics of the observed droplets, we set up FRAP experiments on PrLD-less TDP-43 labelled with 1:10 (dye:protein) Alexa Fluor 488 (PrLD-less TDP-43-Alexa488). Protein phase separation was again induced diluting PrLD-less TDP-43 down to 5 µM in our standard buffer at 150 mM NaCl, as reported in Materials and methods section. Most of the initial condensates at 0 min before FRAP (pre-bleaching) had sizes of 0.5–1.0 µm, with some assemblies starting to interact, reaching a diameter of ~2.0–2.5 μm. A population of larger assemblies (~4.0–4.5 μm) with a non-perfect rounded shape, probably arising from an incomplete coalescence of the initial condensates, appeared 10–15 min after phase separation and was subjected to FRAP experiments (Fig. 2, top panel). The time course of relative fluorescence intensity (RFI) fits well to a double exponential function (Eq. 1). The quantitative analysis showed a significant recovery of RFI (Fig. 2, red curve), indicating that these assemblies possessed liquid characteristics. The fitting analysis revealed a fast recovery phase having a rate constant (*k*_fast_) of ca. 0.09 s^−1^ followed by a second slower one whose rate constant (*k*_slow_) was ca. 0.009 s^−1^ (Fig. 2, red curve). The previously described smaller condensates were also analysed by FRAP (Fig. 2, bottom panel), revealing a low increase of RFI (Fig. 2, green curve), which suggests a low mobility of the PrLD-less TDP-43 molecules inside the assemblies, that is partially due to their weak interaction with the well surface, indirectly favoured by the small size. Even the largest assemblies that undergo fluorescence recovery more efficiently had a few small round assemblies around them that appeared unable to coalesce, suggesting a reduced mobility for these species.

### PrLD-less TDP-43 assemblies caused turbidity in the absence of large structural changes

PrLD-less TDP-43 self-assembly was triggered by incubating the unlabelled protein under final conditions of 5 μM PrLD-less TDP-43, pH 5.5, 0 mM or 150 mM NaCl, 5% (w/v) PEG 8000, 1 mM TCEP, 25 °C (see the Materials and methods for more details) and the process was monitored with turbidimetry, intrinsic fluorescence and far-UV CD.

**Fig. 3:**
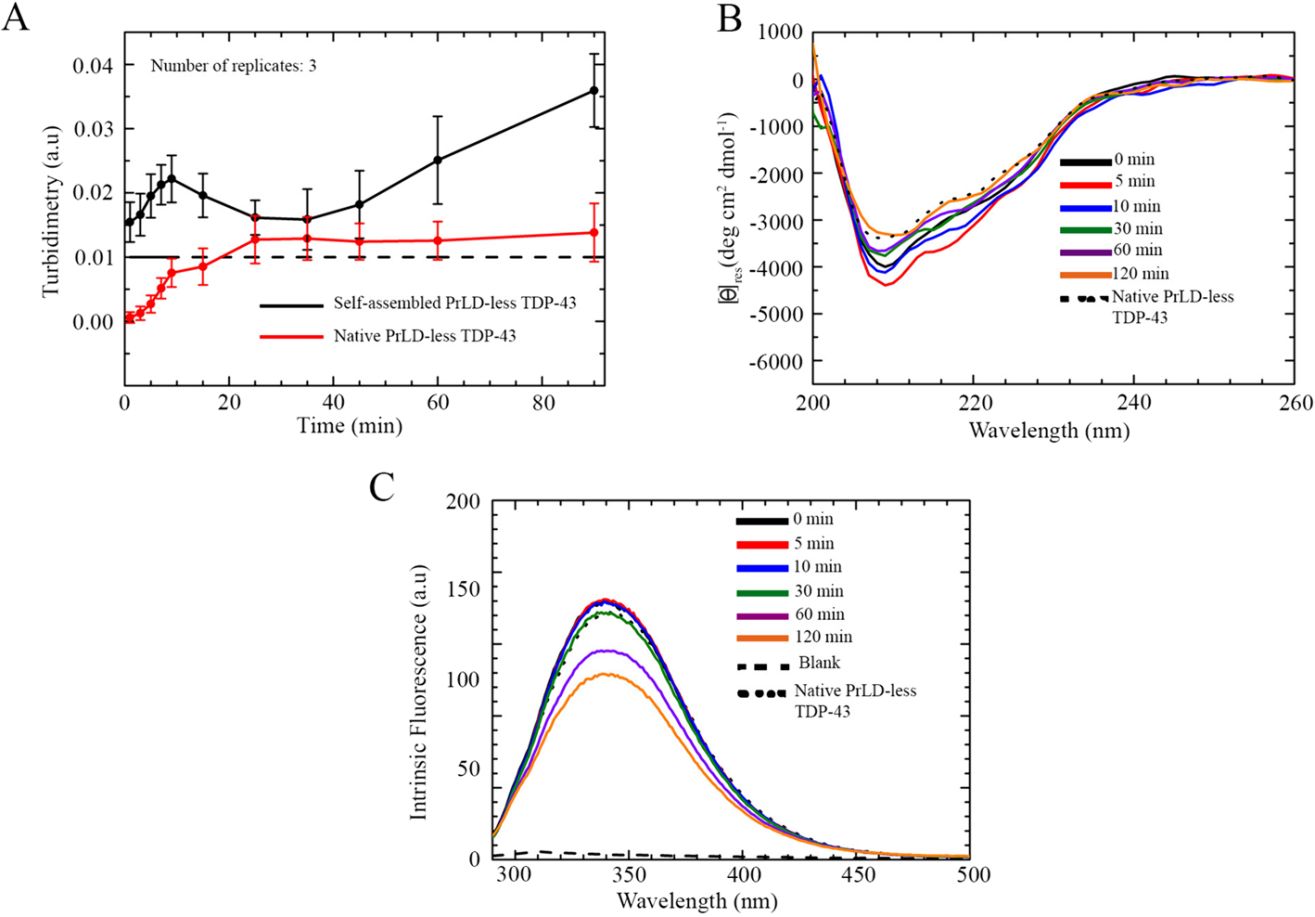
Spectroscopic characterization of PrLD-less TDP-43 self-assembly in 0 mM NaCl. **A** Time course of PrLD-less TDP-43 self-assembly monitored with turbidimetry under native (red) and self-assembly promoting (black) conditions. The number of replicates was 3 (*n* = 3). Error bars: SEM. The dashed line indicates the threshold of turbidimetry above which the signal is significantly high to indicate visible phase separation. **B** Far-UV CD spectra of PrLD-less TDP-43 during self-assembly at the indicated time points. The spectrum of native PrLD-less TDP-43 is also shown. **C** Intrinsic Trp fluorescence spectra of PrLD-less TDP-43 during self-assembly at the indicated time points. The spectrum of native PrLD-less TDP-43 is also shown

At both salt concentrations, the turbidity time course (OD_600_) showed a very small, yet significant, increase after dilution, with a slight increase of OD_600_ values after 5–10 min in 0 mM NaCl, which instead was present to a lower extent in 150 mM NaCl (Fig. 3A, Fig. S4A). This was perceivable by significant scattering above the threshold of turbidimetry indicating the presence of heterogeneous solution. As time elapsed, a further increase of turbidity was detected at both NaCl concentrations, suggesting the formation of bigger assemblies (Fig. 3A, Fig. S4A). However, this later increase was again higher in the absence of salts than in 150 mM NaCl, confirming the confocal fluorescence images showing larger assemblies in the former condition. Considering both turbidimetry and confocal microscopy time course data, PrLD-less TDP-43 phase separated rapidly and formed small species at 0 min (Figs. 1A, B, 3A, Fig. S4A). These species then increased in size without forming the large species that make the solution less cloudy; thus, no decrease in turbidity values was detected, which indeed increased further (Figs. 1A, B, 3A, Fig. S4A). These time courses were in sharp contrast with those detected for full-length TDP-43 using the same experimental conditions and apparatus, in which a first increase of OD_600_, corresponding to the formation of the small spherical assemblies, was followed by a second slower decrease (down to small OD_600_ values), which was associated with formation of the large non-spherical aggregates with irregular structure and absence of coalescence [52]. Moreover, the differences in turbidity values during the time courses (smaller for PrLD-less TDP-43) supported the information obtained with fluorescence microscopy and FRAP experiments, suggesting a probably different state of matter of PrLD-less TDP-43 assemblies. Indeed, they could be soluble in the medium due to their liquid-like state that could scatter light weaker than the gel-like assemblies of full-length TDP-43.

The far-UV CD spectra, recorded immediately after dilution (0 min) in both 0 mM and 150 mM NaCl, showed the absence of major secondary structural changes relative to native PrLD-less TDP-43, as indicated by the persistent negative minima at 222 nm and 208 nm and by the very similar spectra relative to that of native PrLD-less TDP-43 (Fig. 3B, Fig. S4B). As the incubation time elapsed, a slight reduction of the α-helical signals revealed the tendency of the protein to lose partially its initial secondary structures, but without showing any blue shift, typical of a slight growing contribution of the random coil content (Fig. 3B, Fig. S4B). This structural readjustment could be due to the constraints of a high number of protein molecules in a small portion of space, but could also indicate absorption flattening originating from self-assembly, a phenomenon that is more evident in 0 mM NaCl (Fig. 3B, Fig. S4B), where more numerous and slightly larger droplets were present (Fig. 1A, B). At the end of the incubation time, especially in 150 mM NaCl, PrLD-less TDP-43 did not show major secondary structure rearrangements within the droplets (Fig. 3B, Fig. S4B), unlike the full-length protein previously studied [52].

The intrinsic Trp fluorescence spectra of PrLD-less TDP-43 were in agreement with the far-UV CD analysis, since no appreciable shifts were detected for the wavelength of maximum emission, at both NaCl concentrations, compared to the native non-phase separated protein (Fig. 3C, Fig. S4C). Indeed, a major persistent peak at ~ 340–345 nm was appreciable, with a subtle reduction and increase of signal in 0 mM and 150 mM NaCl, respectively. This evidence suggests that the protein did not undergo major structural rearrangements that led to changes of the burial degree of Trp residues within hydrophobic portions of the individual protein molecules (Fig. 3C, Fig. S4C).

### Multi-parametric dependence of PrLD-less self-assembly

**Fig. 4:**
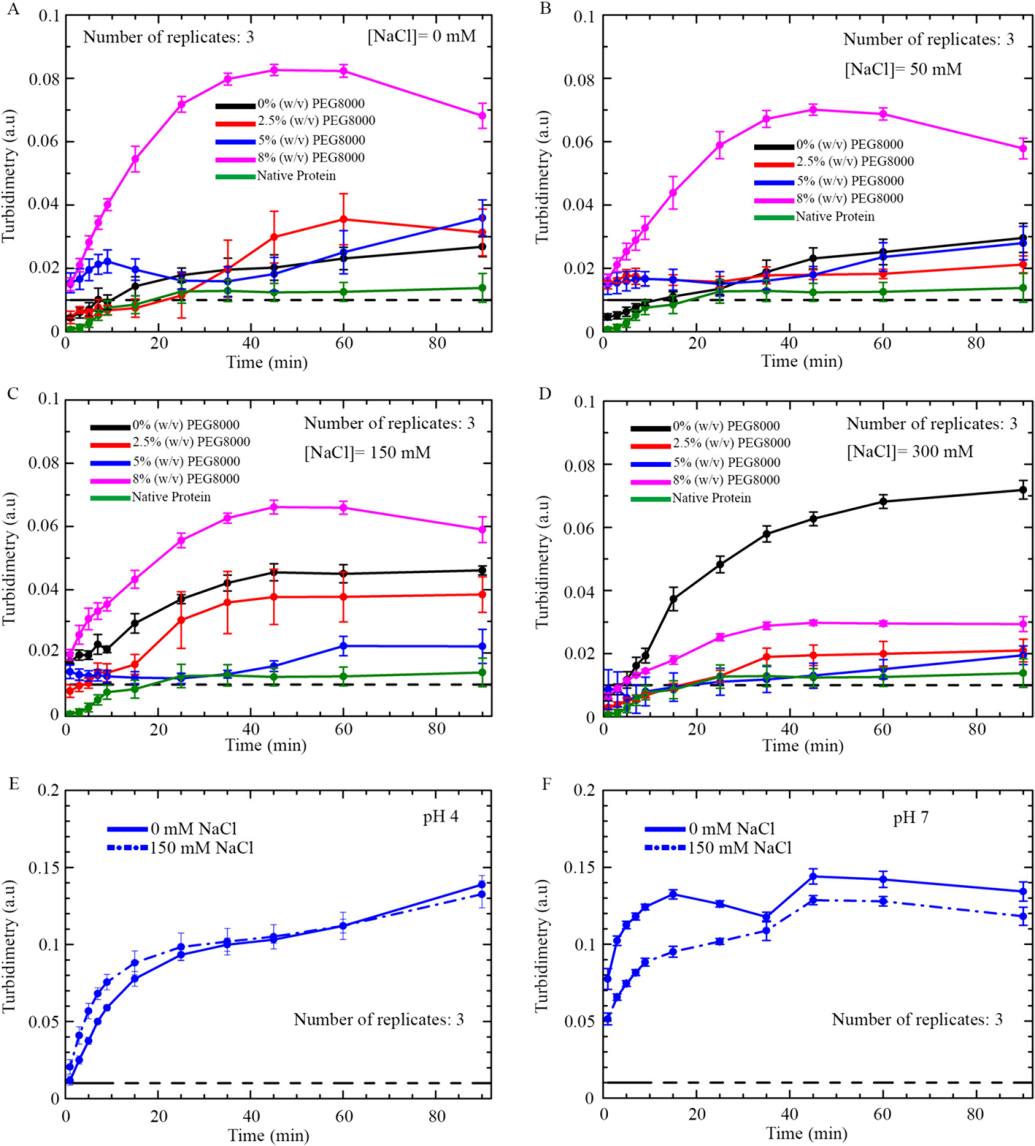
Multi-parametric dependence of PrLD-less TDP-43 self-assembly monitored by turbidimetry. **A**–**D** Fixed parameters were 5 μM PrLD-less TDP-43, pH 5.5, 1 mM TCEP, 25 °C. Final NaCl concentrations were 0 mM (**A**), 50 mM (**B**), 150 mM (**C**) and 300 mM (**D**), each containing 0% (w/v) PEG 8000 (black), 2.5% (w/v) PEG 8000 (red), 5% (w/v) PEG 8000 (blue) or 8% (w/v) PEG 8000 (pink), as indicated in each graph. The native protein was in 20 mM Tris–HCl, pH 8.0 (green). **E**–**F** Fixed parameters were 5 μM PrLD-less TDP-43, 5% (w/v) PEG 8000, 1 mM TCEP, 25 °C, at a final pH of 4.0 (**E**) or 7.0 (**F**). In all panels, final NaCl concentrations were 0 mM (solid line) or 150 mM (dash-dotted line), as indicated in each graph. The number of replicates was 3 (*n* = 3). Error bars: SEM. The dashed line indicates the threshold of turbidimetry above which the signal is significantly high to indicate visible phase separation

Self-assembly of PrLD-less TDP-43 was studied as a function of time, using turbidimetry as an optical probe, and changing NaCl concentration, PEG 8000 concentration, and pH, one by one, while keeping the other parameters constant. Temperature, protein concentration and reducing conditions were constant for all turbidimetry time courses. Conditions were 5 μM PrLD-less TDP-43, 1 mM TCEP, 25 °C, with NaCl concentrations of 0, 50, 150, 300 mM, PEG 8000 concentrations of 0.0, 2.5, 5.0, 8.0% (w/v) and final pH values of 4.0, 5.5 and 7.0. At pH 5.5, corresponding to the isoionic point (pI) of PrLD-less TDP-43, the various turbidity time courses showed a higher degree of self-assembly (perceivable by significant scattering above the threshold of turbidimetry indicating the presence of heterogeneous solution) with the increase of PEG 8000 concentration and with the decrease of NaCl concentration, except at 0% (w/v) PEG 8000 (Fig. 4A–D). Indeed, in the absence of PEG 8000, an increment of salt concentration induced an increase of the OD_600_ values. Interestingly, at the two highest salt concentrations of 150 mM and 300 mM, particularly the latter, the time course in 0% (w/v) PEG 8000 showed a rapid increase of OD_600_ after 10 min, followed by a continuous increase but with a lower rate than the first phase, whereas at other PEG 8000 concentrations, the time courses showed a slight increase after 25–30 min, followed by a plateau phase (Fig. 4).

The dependence of PrLD-less TDP-43 self-assembly on pH is more complex, yet more informative on the electrostatic forces governing LLPS of this protein construct. Turbidity time courses were recorded at pH 4.0, 5.5 and 7.0, in all cases at the two representative NaCl concentrations of 0 and 150 mM. Other conditions were 5 μM PrLD-less TDP-43, 1 mM TCEP, 5.0% (w/v) PEG 8000, 25 °C. PrLD-less TDP-43 self-assembly appeared to be strongly induced at acidic and neutral pH at both NaCl concentrations, as indicated by a remarakble scattering increase over the threshold and arising from the presence of heterophase solution. Moreover, at pH 4.0, which is below the pI of PrLD-less TDP-43, NaCl was found to promote, rather than inhibiting, LLPS of this protein construct, whereas at pH 7.0, above the pI, NaCl was found to inhibit LLPS, similarly to pH 5.5 (Fig. 4E, F).

## Discussion

### PrLD-less TDP-43 undergoes LLPS, unlike the full-length protein

In this report, pure PrLD-less TDP-43 was shown to undergo LLPS under our standard explored conditions (5 μM PrLD-less TDP-43, pH 5.5, 0 mM or 150 mM NaCl, 5% (w/v) PEG 8000, 1 mM TCEP, 25 °C). To be classified as liquid droplets, the assemblies need to satisfy three main criteria: (i) spherical shape, (ii) ability to fuse and (iii) rapid molecular rearrangement [66]. The initial assemblies were round in shape, had a diameter of 0.5–1.0 µm, increased in size and decreased in density as time elapsed, while maintaining their round shape, suggesting they consisted of liquid droplets that fused together with time. Some of the droplets underwent a recovery of RFI after photobleaching to a remarkable extent. RFI recovery was observed mainly in the large droplets that had increased in size more extensively, probably due to their liquid state. Some of the assemblies, particularly the small ones, did not have a considerable RFI recovery, indicating a rather gel-like phase. Even the largest assemblies undergoing RFI recovery had a few small round assemblies around them that appeared unable to coalesce, suggesting a rapid transition from liquid to gel-like phase. The CD and intrinsic fluorescence spectra recorded for the droplet-containing sample were similar to those recorded for the soluble protein before self-assembly, ruling out the occurrence of major structural changes during droplet formation for the protein molecules. Hence, in our experimental settings, PrLD-less TDP-43 assemblies seem to comply with the three main criteria of liquid droplets, in agreement with previous data obtained for the same construct but under different conditions [54].

The results obtained here for PrLD-less TDP-43 are different from those previously obtained in our laboratory for the full-length protein under very similar conditions and with the same experimental apparatus [52]. The condensates of full-length TDP-43 appeared nearly spherical, but did not coalesce and showed limited RFI recovery [52]. In addition, when monitored with turbidimetry, full-length TDP-43 phase separated in a different manner relative to the PrLD-less protein, due to the presence a second exponential phase of turbidity decay corresponding to the formation of larger irregular assemblies that are not formed with the PrLD-less variant.

### Comparison between PrLD-less, PrLD and full-length TDP-43 sheds light on the fundamentals of phase separation of TDP-43

**Fig. 5:**
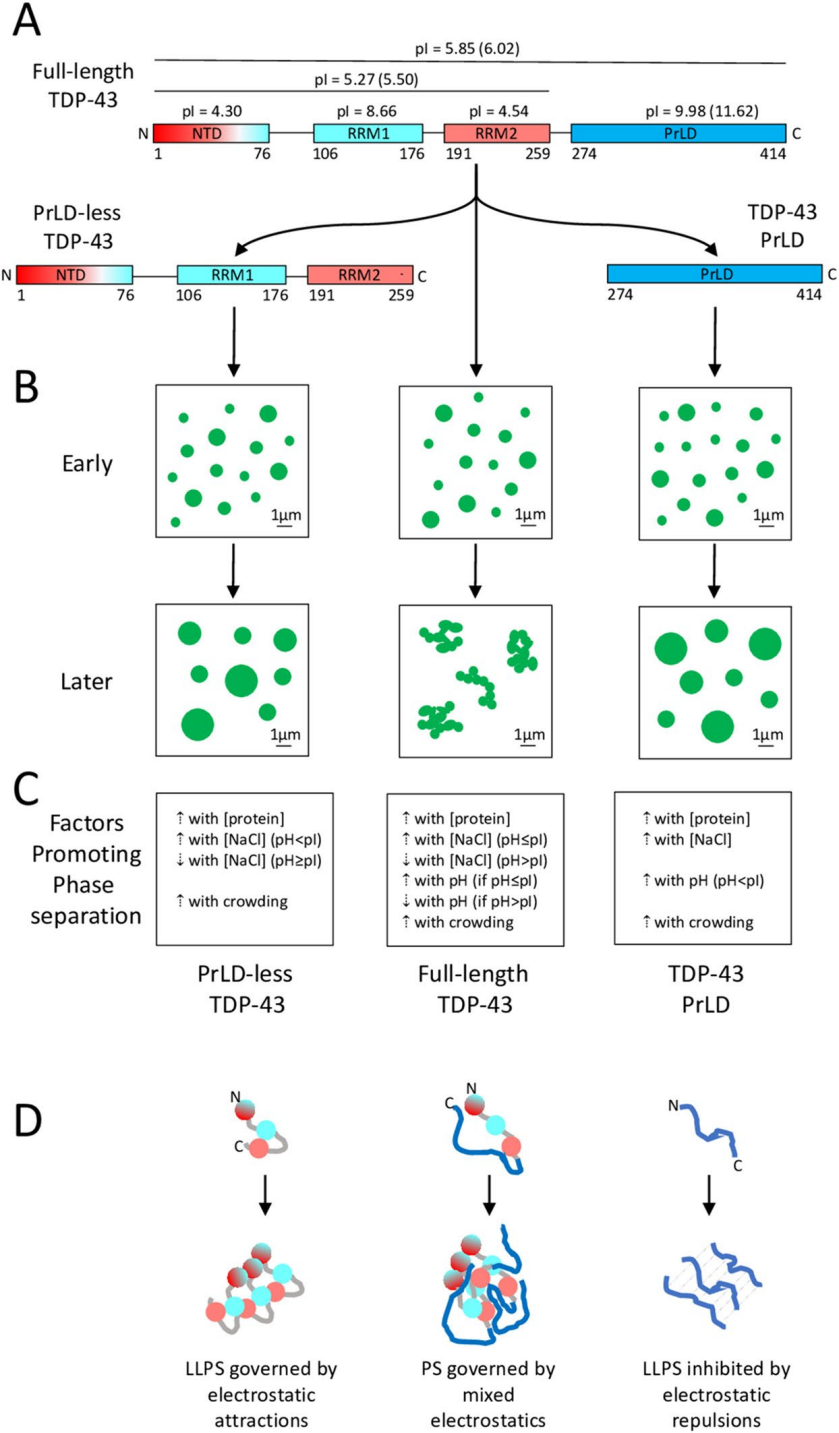
Electrostatic forces governing phase separation of PrLD-less, PrLD and full-length TDP-43. **A** Domains composing full-length (centre), PrLD-less (left) and PrLD (right) TDP-43. Net charges of individual domains at neutral pH are negative if featuring low pI (red), weakly positive with moderately high pI (pale blue) and positive with very high pI (blue). pIs are indicated (pIs with a 6 His-tag in brackets). Numbers below domains indicate residue boundaries. **B** Types of phase separated assemblies observed early (top) and later (bottom) for the TDP-43 constructs. Only PrLD-less (left) and PrLD (right) TDP-43 form genuine liquid droplets. Full-length TDP-43 forms gel-like droplets with limited recovery of RFI after photobleaching that cluster further without fusion (middle). **C** Factors governing phase separation for the three constructs. **D** Schematics of electrostatic forces governing phase separation for the three constructs (colours as in **A**)

The observation that PrLD-less TDP-43 undergoes genuine LLPS, and that it does so with the negative salt dependence observed here at a pH value close to its pI and previously at neutral pH [54], has implications for elucidating the behaviour of the full-length protein, as well. To better discuss this point, we report a scheme with the domain compositions, pIs and net charge at neutral pH (red for negative and blue for positive net charge) for PrLD-less TDP-43, the isolated PrLD and the full-length protein (Fig. 5A). Results on PrLD-less and full-length proteins were obtained in the same laboratory and with the same experimental apparatus and all results summarized in the figure were obtained by multiple investigators as reported in the associated references below, which makes the comparison solid.

LLPS was observed by different investigators working independently of each other for both pure PrLD-less TDP-43 (this work and previously [54]) and pure TDP-43 PrLD [22, 23, 44–47], in all cases observing round and coalescing droplets with high recovery of RFI after photobleaching (Fig. 5B). However, the pH and salt dependences of LLPS for the two protein constructs were found to be different (Fig. 5C). LLPS of the isolated PrLD is promoted by high salt concentrations and becomes more favourable as the pH increases, at least up to 9.0 [23, 45–47]. This has been attributed to the high pI of 9.98 (or 11.62 with the His-tag) for this domain and to the ability of pH values around 9.0 and salts to partially shield the electrostatic repulsions between different PrLD molecules in the droplets [23, 45–47], as shown schematically in Fig. 5D.

By contrast, LLPS of PrLD-less TDP-43 is promoted by salts, but only at acidic pH values lower than its pI of 5.59, as observed here (Fig. 5C). At pH values close or higher than the pI of 5.59, LLPS of PrLD-less TDP-43 is inhibited, rather than promoted, as the salt concentration increases (Fig. 5C), as observed here and previously [54]. This indicates that at neutral pH, LLPS of this protein construct is promoted by electrostatic attractions rather than inhibited by electrostatic repulsions (Fig. 5D). This evidence has a rationale in the argument that LLPS of this construct is driven by the two RRM1 and RRM2 domains and that the NTD domain acts as an enhancing factor due to its oligomerization ability [54]. Indeed, the isolated NTD forms oligomers and then fibrils without unfolding, but not droplets [54], similarly to other globular proteins [67, 68]. An LLPS mainly driven by the RRM1 and RRM2 domains is likely to involve, among other driving forces, electrostatic attractions between these two domains that have pIs of 8.66 and 4.54, respectively, and, therefore, opposite net charges at both the pH of 5.5 and 7.0 studied here and at pH of 7.5 studied previously [54]. Moreover, the LLPS-enhancing factor represented by the folded NTD also dimerizes and oligomerizes through end-to-tail interactions between surface portions with opposite charges [24, 25]. This explains why PrLD-less TDP-43 LLPS occurs more efficiently in the absence of salts when electrostatic attractions are maximized (Fig. 5D). At pH 4.0, all three domains of PrLD-less TDP-43 are positively charged and LLPS is promoted by NaCl, which acts as a shielding factor of the electrostatic repulsions between PrLD-less molecules, as this is the case for the isolated PrLD at all pH values.

What about the full-length protein? First, for the reasons discussed in the previous section, it is more correct to consider a process of liquid–solid or liquid-gel phase separation in this case [25, 48–52, 69] as summarized in our scheme (Fig. 5B). Self-assembly of the full-length protein had a clear pH dependence, with a peak at pH 6.0, which is close to its pI of 5.85 (or 6.02 with the His-tag), and then decreasing in efficiency as the pH changed in both acidic and neutral/basic directions (Fig. 5C) [52]. NaCl was found to promote assembly at acidic pH values, up to 6.0, and then to inhibit it above this pH value (Fig. 5C) [52]. Phase separation of full-length TDP-43 does not appear to be promoted by either the PrLD (we should expect its pH dependence and salt dependence, which is not the case), or by the complementary portion including the first three folded domains (we should expect a peak at pH 5.27 corresponding to its pI and the same positive salt dependence at pH values of 5.5–6.0, which is not again the case). It rather appears that full-length TDP-43 phase-separates as a whole protein due to the cooperation of all domains and interactions between them, where all domains and interactions between them play a role (Fig. 5D). In this complex process, which is maximized in efficiency at the pH corresponding to the pI of the full-length protein rather than those of individual or grouped domains, salts change their effects depending on whether electrostatic attractions or repulsions are dominant. At physiological pH, for example, phase separation is favoured at low salt concentrations, indicating that the droplets are stabilized by electrostatic interactions. At this pH value, only two out of four domains are titrated, providing an explanation as to why attractions dominate. At pH 4.0, by contrast, phase separation is favoured at high salt concentrations, indicating repulsions within the droplets where all four domains are positively charged (pI > 4.0 for all of them). The presence of multiple interactions between four domains that are so diverse in terms of charge, three-dimensional structure and degree of folding, breaks the homogeneity of interactions that is necessary for LLPS and at the same time increases the number of interactors so that the process is rapidly driven to a gel-like or solid phase.

## Conclusions

Overall, the two complementary fragments of TDP-43 corresponding to the PrLD-less protein studied here (this work and [54]) and the isolated PrLD [22, 23, 44–47]) have the ability to undergo LLPS in vitro in the absence of other proteins and RNA molecules. By contrast, the purified full-length protein does not retain this ability [25, 48–52], although it has been reported that it may undergo LLPS in presence of binding partners, such ssDNAs, RNAs and protein chaperones, due to the perturbation of electrostatic and interaction patterns [38, 70–72]. In fact, pure full-length TDP-43 per se rather forms assemblies in a solid or gel-like phase resulting from LSPS rather than LLPS, indicating that the assemblies form as a result of stable inter-domain interactions across the entire sequence rather than forces limited to one of the two halves of the protein that would, otherwise, maintain the original LLPS behaviour of the fragment. The observation that phase separation of full-length TDP-43 occurs most favourably at its corresponding pI and that phase separation of the three protein constructs all have a positive or negative salt dependence that depends on the position of the pH relative to the pI and whether repulsive or attractive electrostatic forces dominate, respectively, suggest that phase separation involves the entire sequence and the set of protein domains that are present in each case, which implies all four domains for the entire full-length protein.

Despite the reiterated difficulty to convert full-length TDP-43 into liquid condensates in vitro in the test tube, TDP-43 has been observed to form liquid membraneless organelles in the presence of RNAs and other proteins in cell cultures, including stress granules containing RNAs and many other proteins such as T cell intracellular antigen 1 [40, 73], stress granule-independent cytosolic droplets containing importin-α and nuclear pore 62 [37], paraspeckles containing a number of proteins and the long non-coding RNA (lncRNA) of nuclear-enriched abundant transcript 1_2 [74], stress nuclear bodies containing the heat shock factor 1 and the scaffold attachment factor B [36], nuclear anisosomes containing heat shock proteins 70 [38], etc. The anisosomes are a particularly interesting case because they do not contain RNA molecules, but chaperones of the heat shock protein 70 family whose ATPase activity is a requirement for the organelles to remain liquid [38]. This indicates how, in the absence of RNAs and/or a complex core architecture provided by other proteins, the droplets require energy to remain liquid and avoid the thermodynamically favourable transition into a gel or solid phase.

### Supplementary Information

Supplementary Material 1.

## Data Availability

The datasets used and analysed during the current study are available from the corresponding author on reasonable request.

## Abbreviations

ALS: Amyotrophic lateral sclerosis
CD: Circular dichroism
CTD: C-Terminal domain
*D*_*h*_: Hydrodynamic diameter
DLS: Dynamic light scattering
DSF: Differential scanning fluorimetry
DTT: Dithiothreitol
FRAP: Fluorescence recovery after photobleaching
FTLD: Frontotemporal lobar degeneration
FTLD-TDP: Frontotemporal lobar degeneration with TDP-43 positive inclusions
GOF: Gain of function
IPTG: Isopropyl β-d-1-thiogalatoside
LATE: Limbic-predominant age-related TDP-43 encephalopathy
LB: Luria–Bertani
LCD: Low-complexity domain
LLPS: Liquid–liquid phase separation
lncRNA: Long non-coding RNA
LSPS: Liquid–solid phase separation
LOF: Loss of function
MBP: Maltose-binding protein
MW: Molecular weight
MWCO: Molecular weight cut-off
NES: Nuclear export signal
Ni–NTA: HisPur Ni–nitrilotriacetic acid
NLS: Nuclear localization signal
NTD: N-Terminal domain
PEG 8000: Poly(ethylene glycol) 8000
pI: Isoionic point
PMSF: Phenylmethanesulfonyl fluoride
PrLD: Prion-like domain
ROIs: Regions of interest
RRMs: RNA recognition motifs
SDS–PAGE: Sodium dodecyl sulphate–polyacrylamide gel electrophoresis
SEC: Size exclusion chromatography
SEM: Standard error of the mean
SUMO: Small ubiquitin-like modifier
TCEP: Tris(2-carboxyethyl)phosphine
TDP-43: TAR DNA-binding protein 43
TMR: Tetramethylrhodamine-5-maleimide

## References

[1] Arai T, Hasegawa M, Akiyama H, Ikeda K, Nonaka T, Mori H, Mann D, Tsuchiya K, Yoshida M, Hashizume Y, Oda T (2006). TDP-43 is a component of ubiquitin-positive tau-negative in-clusions in frontotemporal lobar degeneration and amyotrophic lateral sclerosis. Biochem Biophys Res Commun.

[2] Neumann M, Sampathu DM, Kwong LK, Truax AC, Micsenyi MC, Chou TT, Bruce J, Schuck T, Grossman M, Clark CM, McCluskey LF, Miller BL, Masliah E, Mackenzie IR, Feldman H, Feiden W, Kretzschmar HA, Trojanowski JQ, Lee VM (2006). Ubiquitinated TDP-43 in frontotemporal lobar degeneration and amyotrophic lateral sclerosis. Science.

[3] Neumann M, Mackenzie IRA (2019). Review: neuropathology of non-tau frontotemporal lobar degeneration. Neuropathol Appl Neurobiol.

[4] Robberecht W, Philips T (2013). The changing scene of amyotrophic lateral sclerosis. Nat Rev Neurosci.

[5] Nelson PT, Dickson DW, Trojanowski JQ, Jack CR, Boyle PA, Arfanakis K, Rademakers R, Alafuzoff I, Attems J, Brayne C, Coyle-Gilchrist ITS, Chui HC, Fardo DW, Flanagan ME, Halliday G, Hokkanen SRK, Hunter S, Jicha GA, Katsumata Y, Kawas CH, Keene CD, Kovacs GG, Kukull WA, Levey AI, Makkinejad N, Montine TJ, Murayama S, Murray ME, Nag S, Rissman RA, Seeley WW, Sperling RA, White CL, Yu L, Schneider JA (2019). Limbic-predominant age-related TDP-43 encephalopathy (LATE): consensus working group report. Brain.

[6] Arseni D, Chen R, Murzin AG, Peak-Chew SY, Garringer HJ, Newell KL, Kametani F, Robinson AC, Vidal R, Ghetti B, Hasegawa M, Ryskeldi-Falcon B (2023). TDP-43 forms amyloid filaments with a distinct fold in type A FTLD-TDP. Nature.

[7] Seyfried NT, Gozal YM, Dammer EB, Xia Q, Duong DM, Cheng D, Lah JJ, Levey AI, Peng J (2010). Multiplex SILAC analysis of a cellular TDP-43 proteinopathy model reveals protein inclusions associated with SUMOylation and diverse polyubiquitin chains. Mol Cell Proteomics.

[8] Cohen TJ, Hwang AW, Restrepo CR, Yuan CX, Trojanowski JQ, Lee VM (2015). An acetylation switch controls TDP-43 function and aggregation propensity. Nat Commun.

[9] Polymenidou M, Cleveland DW (2011). The seeds of neurodegeneration: prion-like spreading in ALS. Cell.

[10] Ravits JM, La Spada AR (2009). ALS motor phenotype heterogeneity, focality, and spread: deconstructing motor neuron degeneration. Neurology.

[11] Igaz LM, Kwong LK, Xu Y, Truax AC, Uryu K, Neumann M, Clark CM, Elman LB, Miller BL, Grossman M, McCluskey LF, Trojanowski JQ, Lee VM (2008). Enrichment of C-terminal fragments in TAR DNA-binding protein-43 cytoplasmic inclusions in brain but not in spinal cord of frontotemporal lobar degeneration and amyotrophic lateral sclerosis. Am J Pathol.

[12] Cohen TJ, Lee VMY, Trojanowski JQ (2011). TDP-43 functions and pathogenic mechanisms implicated in TDP-43 proteinopathies. Trends Mol Med.

[13] Halliday G, Bigio EH, Cairns NJ, Neumann M, Mackenzie IR, Mann DM (2012). Mechanisms of disease in frontotemporal lobar degeneration: gain of function versus loss of function effects. Acta Neuropathol.

[14] Lukavsky PJ, Daujotyte D, Tollervey JR, Ule J, Stuani C, Buratti E, Baralle FE, Damberger FF, Allain FH (2013). Molecular basis of UG-rich RNA recognition by the human splicing factor TDP-43. Nat Struct Mol Biol.

[15] Cascella R, Capitini C, Fani G, Dobson CM, Cecchi C, Chiti F (2016). Quantification of the relative contributions of loss-of-function and gain-of-function mechanisms in TAR DNA-binding protein 43 (TDP-43) Proteinopathies. J Biol Chem.

[16] De Marchi F, Franjkic T, Schito P, Russo T, Nimac J, Chami AA, Mele A, Vidatic L, Kriz J, Julien JP, Apic G, Russell RB, Rogelj B, Cannon JR, Baralle M, Agosta F, Hecimovic S, Mazzini L, Buratti E, Munitic I (2023). Emerging trends in the field of inflammation and proteinopathy in ALS/FTD spectrum disorder. Biomedicines.

[17] Nakashima-Yasuda H, Uryu K, Robinson J, Xie SX, Hurtig H, Duda JE, Arnold SE, Siderowf A, Grossman M, Leverenz JB, Woltjer R, Lopez OL, Hamilton R, Tsuang DW, Galasko D, Masliah E, Kaye J, Clark CM, Montine TJ, Lee VMY, Trojanowski JQ (2007). Co-morbidity of TDP-43 proteinopathy in Lewy body related diseases. Acta Neuropathol.

[18] Amador-Ortiz C, Lin WL, Ahmed Z, Personett D, Davies P, Duara R, Graff-Radford NR, Hutton ML, Dickson DW (2007). TDP-43 immunoreactivity in hippocampal sclerosis and Alzheimer’s disease. Ann Neurol.

[19] Schwab C, Arai T, Hasegawa M, Yu S, McGeer PL (2008). Colocalization of transactivation-responsive DNA-binding protein 43 and huntingtin in inclusions of Huntington disease. J Neuropathol Exp Neurol.

[20] Miguelez-Rodriguez A, Santos-Juanes J, Vicente-Etxenausia I, Perez de Heredia-Goñi K, Garcia B, Quiros LM, Lorente-Gea L, Guerra-Merino I, Aguirre JJ, Fernandez-Vega I (2018). Brains with sporadic Creutzfeldt-Jakob disease and copathology showed a prolonged end-stage of disease. J Clin Pathol.

[21] Mez J, Daneshvar DH, Kiernan PT, Abdolmohammadi B, Alvarez VE, Huber BR, Alosco ML, Solomon TM, Nowinski CJ, McHale L, Cormier KA, Kubilus CA, Martin BM, Murphy L, Baugh CM, Montenigro PH, Chaisson CE, Tripodis Y, Kowall NW, Weuve J, McClean MD, Cantu RC, Goldstein LE, Katz DI, Stern RA, Stein TD, McKee AC (2017). Clinicopathological evaluation of chronic traumatic encephalopathy in players of american football. JAMA.

[22] Conicella A, Zerze G, Mittal J, Fawzi N (2016). ALS mutations disrupt phase separation mediated by α-helical structure in the TDP-43 low-complexity C-terminal domain. Structure.

[23] Li HR, Chen TC, Hsiao CL, Shi L, Chou CY, Huang JR (2018). The physical forces mediating self-association and phase-separation in the C-terminal domain of TDP-43. Biochem Biophys Acta Proteins Proteom.

[24] Afroz T, Hock EM, Ernst P, Foglieni C, Jambeau M, Gilhespy LAB, Laferriere F, Maniecka Z, Plückthun A, Mittl P, Paganetti P, Allain FHT, Polymenidou M (2017). Functional and dynamic polymerization of the ALS-linked protein TDP-43 antagonizes its pathologic aggregation. Nat Commun.

[25] Wang A, Conicella AE, Schmidt HB, Martin EW, Rhoads SN, Reeb AN, Nourse A, Ramirez Montero D, Ryan VH, Rohatgi R, Shewmaker F, Naik MT, Mittag T, Ayala YM, Fawzi NL (2018). A single N-terminal phosphomimic disrupts TDP-43 polymerization, phase separation, and RNA splicing. EMBO J.

[26] Ayala YM, Zago P, D'Ambrogio A, Xu YF, Petrucelli L, Buratti E, Baralle FE (2008). Structural determinants of the cellular localization and shuttling of TDP-43. J Cell Sci.

[27] Shiina Y, Arima K, Tabunoki H, Satoh J (2010). TDP-43 dimerizes in human cells in culture. Cell Mol Neurobiol.

[28] Chang CK, Wu TH, Wu CY, Chiang MH, Toh EK, Hsu YC, Lin KF, Liao YH, Huang TH, Huang JJ (2012). The N-terminus of TDP-43 promotes its oligomerization and enhances DNA binding affinity. Biochem Biophys Res Commun.

[29] Zhang YJ, Caulfield T, Xu YF, Gendron TF, Hubbard J, Stetler C, Sasaguri H, Whitelaw EC, Cai S, Lee WC, Petrucelli L (2013). The dual functions of the extreme N-terminus of TDP-43 in regulating its biological activity and inclusion formation. Hum Mol Genet.

[30] Wang YT, Kuo PH, Chiang CH, Liang JR, Chen YR, Wang S, Shen JC, Yuan HS (2013). The truncated C-terminal RNA recognition motif of TDP-43 protein plays a key role in forming proteinaceous aggregates. J Biol Chem.

[31] Mompeán M, Romano V, Pantoja-Uceda D, Stuani C, Baralle FE, Buratti E, Laurents DV (2016). The TDP-43 N-terminal domain structure at high resolution. FEBS J.

[32] Moisse K, Volkening K, Leystra-Lantz C, Welch I, Hill T, Strong MJ (2009). Divergent patterns of cytosolic TDP-43 and neuronal progranulin expression following axotomy: implications for TDP-43 in the physiological response to neuronal injury. Brain Res.

[33] Colombrita C, Zennaro E, Fallini C, Weber M, Sommacal A, Buratti E, Silani V, Ratti A (2009). TDP-43 is recruited to stress granules in conditions of oxidative insult. J Neurochem.

[34] Dammer EB, Fallini C, Gozal YM, Duong DM, Rossoll W, Xu P, Lah JJ, Levey AI, Peng J, Bassell GJ, Seyfried NT (2012). Coaggregation of RNA-binding proteins in a model of TDP-43 proteinopathy with selective RGG motif methylation and a role for RRM1 ubiquitination. PLoS ONE.

[35] Nishimoto Y, Nakagawa S, Hirose T, Okano HJ, Takao M, Shibata S, Suyama S, Kuwako KI, Imai T, Murayama S (2013). The long non-coding RNA nuclear-enriched abundant transcript 1_2 induces paraspeckle formation in the motor neuron during the early phase of amyotrophic lateral sclerosis. Mol Brain.

[36] Udan-Johns M, Bengoechea R, Bell S, Shao J, Diamond MI, True HL, Weihl CC, Baloh RH (2014). Prion-like nuclear aggregation of TDP-43 during heat shock is regulated by HSP40/70 chaperones. Hum Mol Genetics.

[37] Gasset-Rosa F, Lu S, Yu H, Chen C, Melamed Z, Guo L, Shorter J, Da Cruz S, Cleveland D (2019). Cytoplasmic TDP-43 de-mixing independent of stress granules drives inhibition of nuclear import, loss of nuclear TDP-43, and cell death. Neuron.

[38] Yu H, Lu S, Gasior K, Singh D, Vazquez-Sanchez S, Tapia O, Toprani D, Beccari MS, Yates JR, Da Cruz S, Newby JM, Lafarga M, Gladfelter AS, Villa E, Cleveland DW (2021). HSP70 chaperones RNA-free TDP-43 into anisotropic intranuclear liquid spherical shells. Science.

[39] Carey JL, Guo L (2022). Liquid-liquid phase separation of TDP-43 and FUS in physiology and pathology of neurodegenerative diseases. Front Mol Biosci.

[40] Chen Y, Cohen T (2019). Aggregation of the nucleic acid–binding protein TDP-43 occurs via distinct routes that are coordinated with stress granule formation. J Biol Chem.

[41] Hans F, Glasebach H, Kahle P (2020). Multiple distinct pathways lead to hyperubiquitylated insoluble TDP-43 protein independent of its translocation into stress granules. J Biol Chem.

[42] Ratti A, Gumina V, Lenzi P, Bossolasco P, Fulceri F, Volpe C, Bardelli D, Pregnolato F, Maraschi A, Fornai F, Silani V, Colombrita C (2020). Chronic stress induces formation of stress granules and pathological TDP-43 aggregates in human ALS fibroblasts and iPSC-motoneurons. Neurobiol Dis.

[43] Cascella R, Bigi A, Riffert DG, Gagliani MC, Ermini E, Moretti M, Cortese K, Cecchi C, Chiti F (2022). A quantitative biology approach correlates neuronal toxicity with the largest inclusions of TDP-43. Sci Adv.

[44] Choi KJ, Tsoi PS, Moosa MM, Paulucci-Holthauzen A, Liao SJ, Ferreon JC, Ferreon ACM (2018). A chemical chaperone decouples TDP-43 disordered domain phase separation from fibrillation. Biochemistry.

[45] Babinchak WM, Haider R, Dumm BK, Sarkar P, Surewicz K, Choi JK, Surewicz WK (2019). The role of liquid–liquid phase separation in aggregation of the TDP-43 low-complexity domain. J Biol Chem.

[46] Pakravan D, Michiels E, Bratek-Skicki A, De Decker M, Van Lindt J, Alsteens D, Derclaye S, Van Damme P, Schymkowitz J, Rousseau F, Tompa P, Van Den Bosch L (2021). Liquid-liquid phase separation enhances TDP-43 LCD aggregation but delays seeded aggregation. Biomolecules.

[47] Garg DK, Bhat R (2022). Modulation of assembly of TDP-43 low-complexity domain by heparin: From droplets to amyloid fibrils. Biophys J.

[48] Molliex A, Temirov J, Le J, Coughlin M, Kanagaraj AP, Kim HJ, Mittag T, Taylor JP (2015). Phase separation by low complexity domains promotes stress granule assembly and drives pathological fibrillization. Cell.

[49] Sun Y, Medina Cruz A, Hadley KC, Galant NJ, Law R, Vernon RM, Morris VK, Robertson J, Chakrabartty A (2019). Physiologically important electrolytes as regulators of TDP-43 aggregation and droplet-phase behavior. Biochemistry.

[50] Krainer G, Welsh TJ, Joseph JA, Espinosa JR, Wittmann S, de Csilléry E, Sridhar A, Toprakcioglu Z, Gudiškytė G, Czekalska MA, Arter WE, Guillén-Boixet J, Franzmann TM, Qamar S, George-Hyslop PS, Hyman AA, Collepardo-Guevara R, Alberti S, Knowles TPJ (2021). Reentrant liquid condensate phase of proteins is stabilized by hydrophobic and non-ionic interactions. Nat Commun.

[51] Gruijs Silva LA, Simonetti F, Hutten S, Riemenschneider H, Sternburg EL, Pietrek LM, Gebel J, Dötsch V, Edbauer D, Hummer G, Stelzl LS, Dormann D (2022). Disease-linked TDP-43 hyperphosphorylation suppresses TDP-43 condensation and aggregation. EMBO J.

[52] Staderini T, Bigi A, Mongiello D, Chiti F (2022). Biophysical characterization of full length TAR DNA-binding protein (TDP-43) phase separation. Protein Sci.

[53] McGurk L, Gomes E, Guo L, Mojsilovic-Petrovic J, Tran V, Kalb RG, Shorter J, Bonini NM (2018). Poly(ADP-Ribose) prevents pathological phase separation of TDP-43 by promoting liquid demixing and stress granule localization. Mol Cell.

[54] Carter GC, Hsiung CH, Simpson L, Yang H, Zhang X (2021). N-terminal domain of TDP43 enhances liquid-liquid phase separation of globular proteins. J Mol Biol.

[55] Wright GSA, Watanabe TF, Amporndanai K, Plotkin SS, Cashman NR, Antonyuk SV, Hasnain SS (2020). Purification and structural characterization of aggregation-prone human TDP-43 involved in neurodegenerative diseases. iScience.

[56] Ballou DP, Benore M, Ninfa AJ (2008). Fundamental laboratory approaches for biochemistry and biotechnology.

[57] Meyer RK, Lustig A, Oesch B, Fatzer R, Zurbriggen A, Vandevelde M (2000). A monomer-dimer equilibrium of a cellular prion protein (PrPC) not observed with recombinant PrP. J Biol Chem.

[58] Beloti LL, Costa BZ, Toledo MA, Santos CA, Crucello A, Fávaro MT, Santiago AS, Mendes JS, Marsaioli AJ, Souza AP (2013). A novel and enantioselective epoxide hydrolase from *Aspergillus brasiliensis* CCT 1435: purification and characterization. Protein Expr Purif.

[59] Rastogi N, Zarin S, Alam A, Konduru GV, Manjunath P, Mishra A, Kumar S, Nagarajaram HA, Hasnain SE, Ehtesham NZ (2023). Structural and biophysical properties of therapeutically important proteins Rv1509 and Rv2231A of *Mycobacterium tuberculosis*. Int J Biol Macromol.

[60] Vivoli Vega M, Nigro A, Luti S, Capitini C, Fani G, Gonnelli L, Boscaro F, Chiti F (2019). Isolation and characterization of soluble human full-length TDP-43 associated with neurodegeneration. FASEB J.

[61] Zacco E, Graña-Montes R, Martin SR, de Groot NS, Alfano C, Tartaglia GG, Pastore A (2019). RNA as a key factor in driving or preventing self-assembly of the TAR DNA-binding protein 43. J Mol Biol.

[62] Kuo PH, Doudeva LG, Wang YT, Shen CK, Yuan HS (2009). Structural insights into TDP-43 in nucleic-acid binding and domain interactions. Nucleic Acids Res.

[63] Chiang CH, Grauffel C, Wu LS, Kuo PH, Doudeva LG, Lim C, Shen CK, Yuan HS (2016). Structural analysis of disease-related TDP-43 D169G mutation: linking enhanced stability and caspase cleavage efficiency to protein accumulation. Sci Rep.

[64] Lackowicz JR (2006). Principles of fluorescence spectroscopy.

[65] Comert F, Dubin PL (2017). Liquid-liquid and liquid-solid phase sepa- ration in protein-polyelectrolyte systems. Adv Colloid Interface Sci.

[66] Gopal PP, Nirschl JJ, Klinman E, Holzbaur EL (2017). Amyotrophic lateral sclerosis-linked mutations increase the viscosity of liquid-like TDP-43 RNP granules in neurons. Proc Natl Acad Sci U S A.

[67] Soldi G, Bemporad F, Torrassa S, Relini A, Ramazzotti M, Taddei N, Chiti F (2005). Amyloid formation of a protein in the absence of initial unfolding and destabilization of the native state. Biophys J.

[68] De Simone A, Dhulesia A, Soldi G, Vendruscolo M, Hsu ST, Chiti F, Dobson CM (2011). Experimental free energy surfaces reveal the mechanisms of maintenance of protein solubility. Proc Natl Acad Sci U S A.

[69] Capitini C, Conti S, Perni M, Guidi F, Cascella R, De Poli A, Penco A, Relini A, Cecchi C, Chiti F (2014). TDP-43 inclusion bodies formed in bacteria are structurally amorphous, non-amyloid and inherently toxic to neuroblastoma cells. PLoS ONE.

[70] Gu J, Wang C, Hu R, Li Y, Zhang S, Sun Y, Wang Q, Li D, Fang Y, Liu C (2021). Hsp70 chaperones TDP-43 in dynamic, liquid-like phase and prevents it from amyloid aggregation. Cell Res.

[71] Lu S, Hu J, Arogundade OA, Goginashvili A, Vazquez-Sanchez S, Diedrich JK, Gu J, Blum J, Oung S, Ye Q, Yu H, Ravits J, Liu C, Yates JR, Cleveland DW (2022). Heat-shock chaperone HSPB1 regulates cytoplasmic TDP-43 phase separation and liquid-to-gel transition. Nat Cell Biol.

[72] Maharana S, Wang J, Papadopoulos DK, Richter D, Pozniakovsky A, Poser I, Bickle M, Rizk S, Guillén-Boixet J, Franzmann TM, Jahnel M, Marrone L, Chang YT, Sterneckert J, Tomancak P, Hyman AA, Alberti S (2018). RNA buffers the phase separation behavior of prion-like RNA binding proteins. Science.

[73] Chew J, Cook C, Gendron TF, Jansen-West K, Del Rosso G, Daughrity LM, Castanedes-Casey M, Kurti A, Stankowski JN, Disney MD, Rothstein JD, Dickson DW, Fryer JD, Zhang YJ, Petrucelli L (2019). Aberrant deposition of stress granule-resident proteins linked to C9orf72-associated TDP-43 proteinopathy. Mol Neurodegener.

[74] Wang C, Duan Y, Duan G, Wang Q, Zhang K, Deng X, Qian B, Gu J, Ma Z, Zhang S, Guo L, Liu C, Fang Y (2020). Stress induces dynamic, cytotoxicity-antagonizing TDP-43 nuclear bodies via paraspeckle LncRNA NEAT1-mediated liquid-liquid phase separation. Mol Cell.

