# Supplementary material for: Biophysical characterization of the phase separation of TDP-43 devoid of the C-terminal domain
Source: Cell Mol Biol Lett. 2024 Jul 13;29:104. doi: 10.1186/s11658-024-00615-4 (PMC11245819; doi:10.1186/s11658-024-00615-4)
Supplement: Supplementary file 1 — Supplementary Material 1. [file 11658_2024_615_MOESM1_ESM.docx]

**Supplementary Information**

**
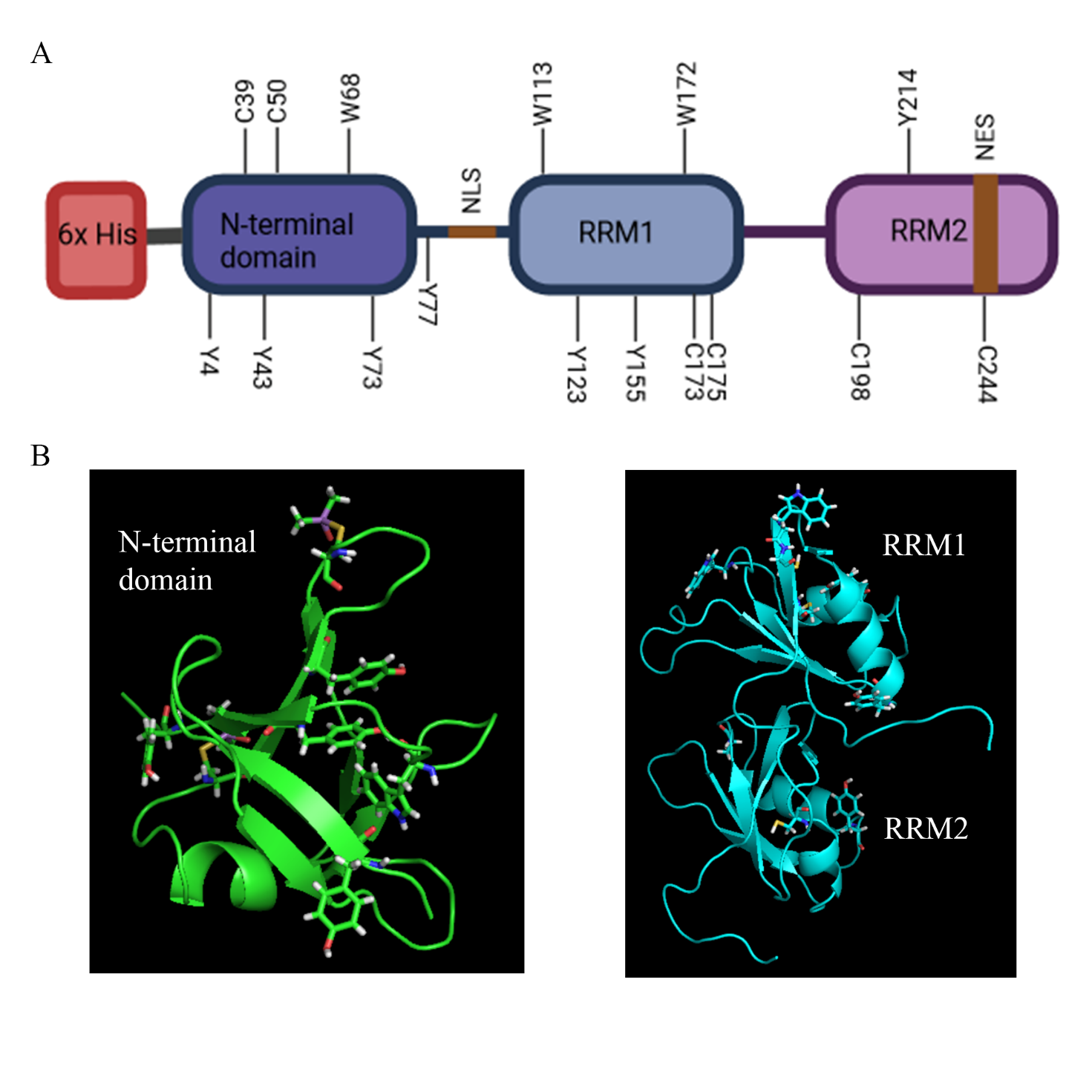
**

**Fig. S1 Schematic representation of PrLD-less TDP-43 and its NTD, RRM1, RRM2 domains.** (**A**) PrLD-less TDP-43 contains the N-terminal domain (NTD_1-76_), a nuclear localization signal (NLS_82-98_), two RNA recognition motifs (RRM1_106–176_ and RRM2_191–259_) and a nuclear exportation signal (NES_239-250_). 16 residues (3 Trp, 7 Tyr and 6 Cys) have been highlighted to provide further structural information. Created with BioRender.com. (**B**) Ribbon diagram structures of the NTD (PDB code: 5MDI) and RRM domains (PDB code: 4BS2) with the Cys, Trp and Tyr residues highlighted as “ball-and-sticks” [11;22]. Generated with PyMOL (<http://www.pymol.org/>)


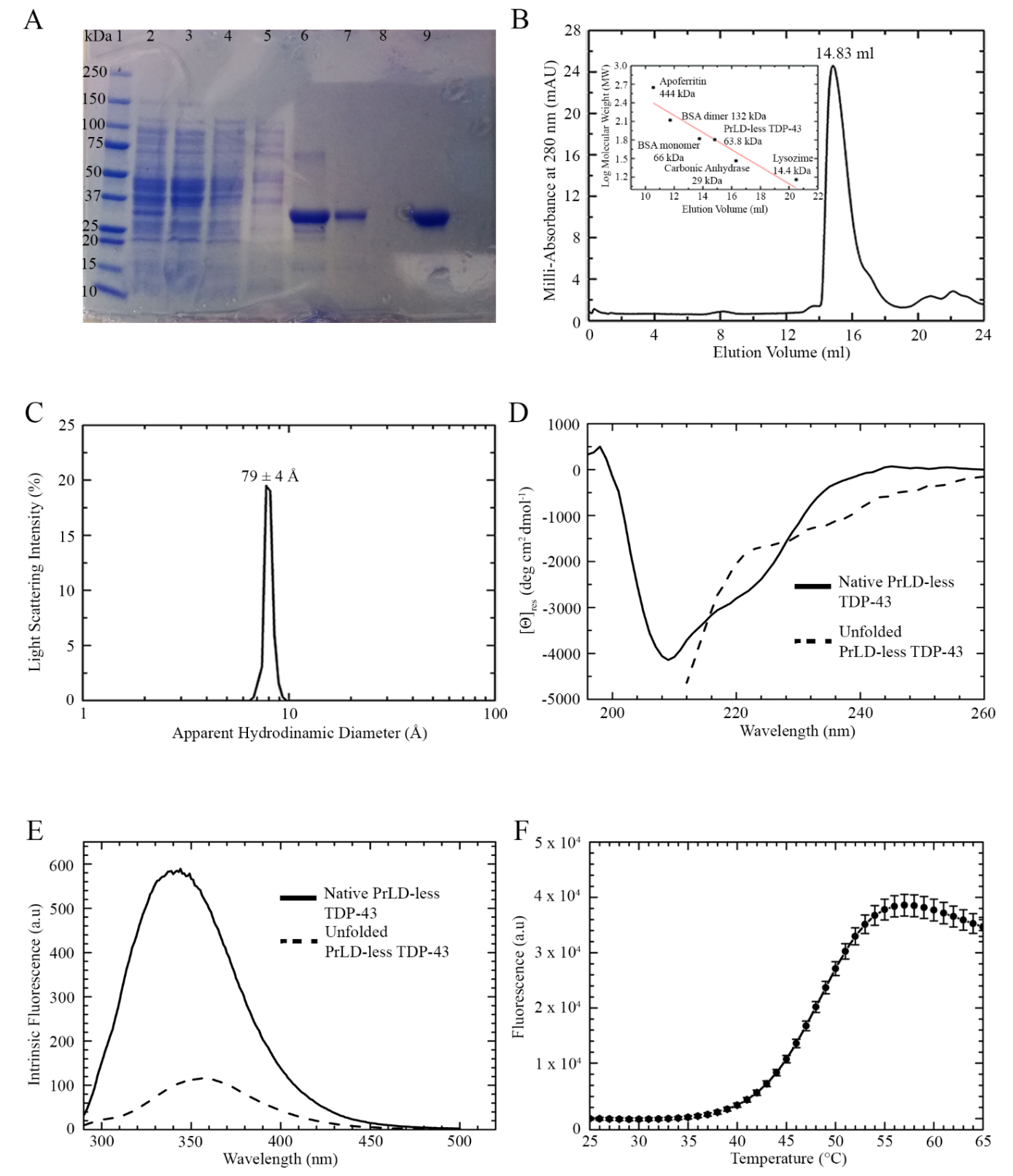


**Fig. S2 Quality control of purified PrLD-less TDP-43.** (**A**) SDS-PAGE of expressed and purified PrLD-less TDP-43. Lane 1: protein ladder. Lane 2: after cell lysis and streptomycin treatment for DNA precipitation. Lanes 3-6: flow through, wash, first and second elution steps of Ni-NTA chromatography. Lanes 7-8: fraction corresponding to the first and second peaks eluted with DEAE chromatography. Lane 9: Main peak eluting from SEC. (**B**) Analytical SEC of PrLD-less TDP-43 with the calibration curve generated with the indicated molecular mass standards ranging from 14.4 kDa to 443 kDa. (**C**) Particle size distribution of PrLD-less TDP-43 determined with DLS. (**D**) Far-UV CD spectrum of native and unfolded PrLD-less TDP-43. (**E**) Intrinsic fluorescence spectrum of native and unfolded PrLD-less TDP-43 (excitation at 280 nm). a.u, arbitrary units. (**F**) DSF curve of PrLD-less TDP-43, obtained with Sypro Orange as a fluorophore. The line indicates the transition from the folded to the unfolded state (n=3). Error bars: standard error of the mean (SEM).


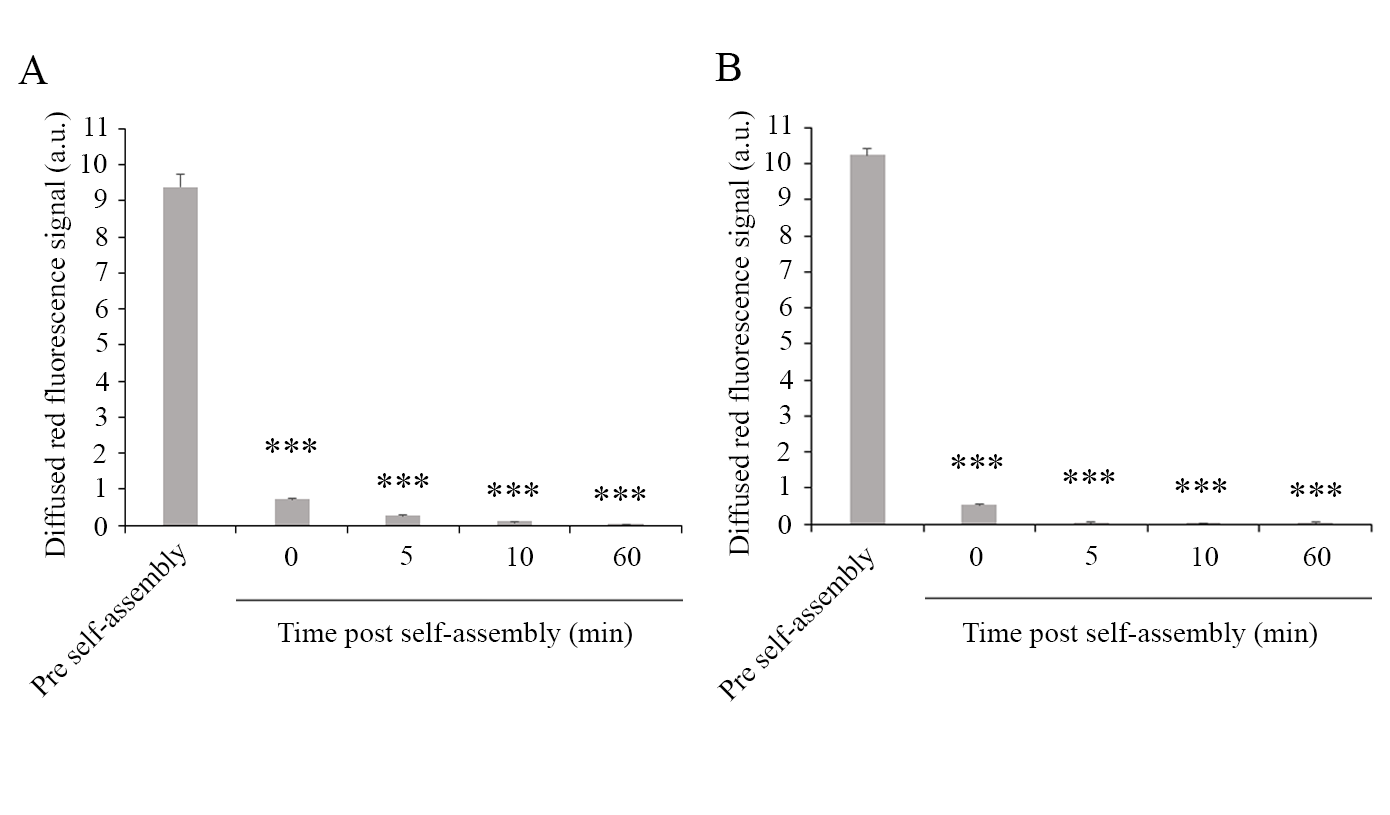


**Fig. S3**  **Analysis of PrLD-less TDP-43 partitioning into the assemblies.** Diffused red fluorescence signal in the background (outside the droplets) arising from PrLD-less TDP-43-TMR before and after self-assembly at time 0, 5, 10, 60 min, referring to Fig. 1A,B. Experimental errors are S.E.M. (n=3). Samples were analyzed by Student’s t-test relative to pre-self-assembly (*** P<0.001).


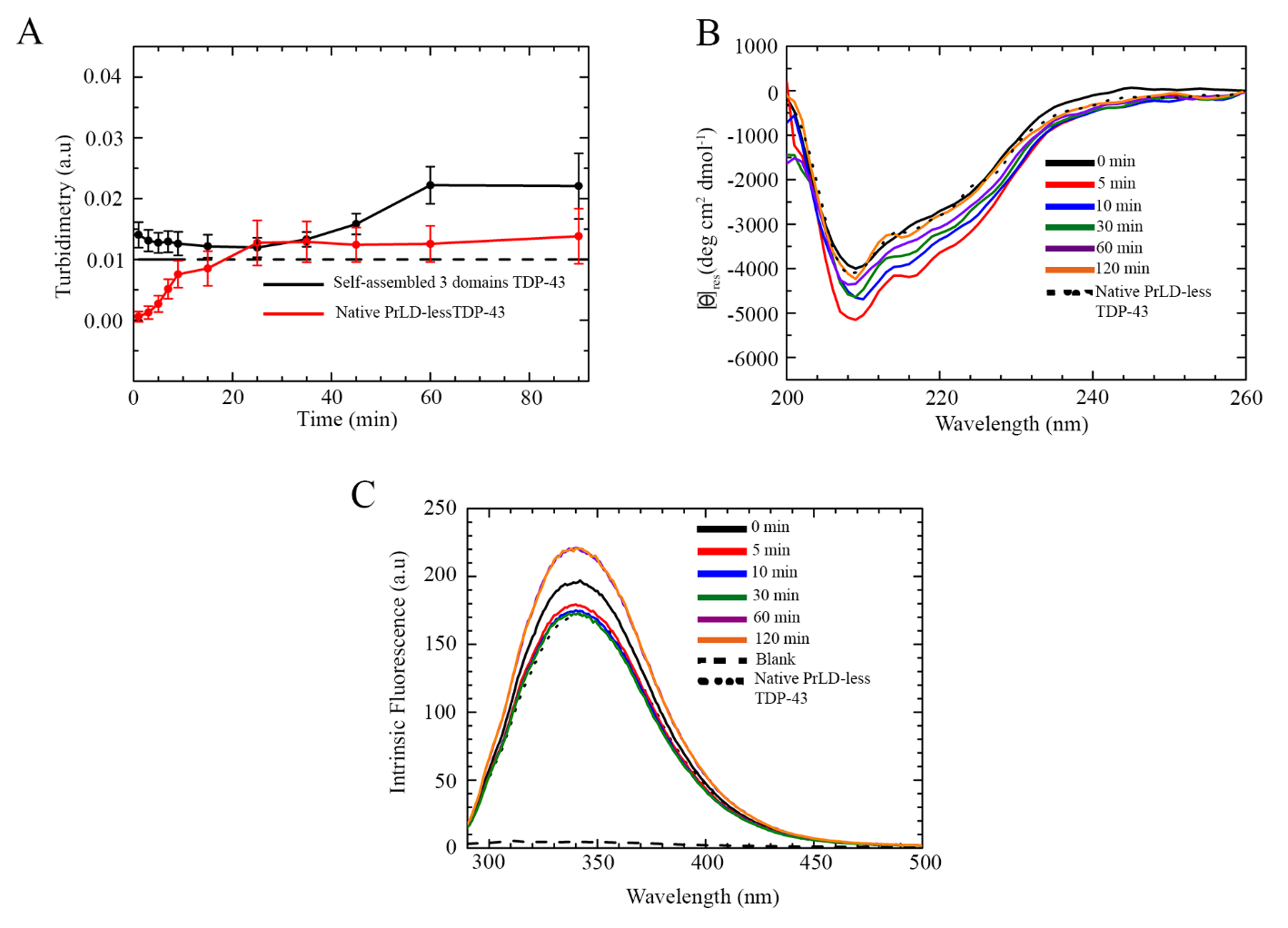


**Fig. S4** **Spectroscopic characterization of PrLD-less TDP-43 self-assembly in 150 mM NaCl.** (**A**) Time course of PrLD-less TDP-43 self-assembly monitored with turbidimetry under native (red) and self-assembly promoting (black) conditions. Number of replicates was 3 (n=3). Error bars: SEM. (**B**) Far-UV CD spectra of PrLD-less TDP-43 during self-assembly at the indicated time points. The spectrum of native PrLD-less TDP-43 is also shown. (**C**) Intrinsic Trp fluorescence spectra of PrLD-less TDP-43 during self-assembly at the indicated time points. The spectrum of native PrLD-less TDP-43 is also shown. Error bars: SEM. The dashed line indicates the threshold of turbidimetry above which the signal is significantly high to indicate visible phase separation.
